# Auditory midbrain coding of statistical learning that results from discontinuous sensory stimulation

**DOI:** 10.1371/journal.pbio.2005114

**Published:** 2018-07-26

**Authors:** Hugo Cruces-Solís, Zhizi Jing, Olga Babaev, Jonathan Rubin, Burak Gür, Dilja Krueger-Burg, Nicola Strenzke, Livia de Hoz

**Affiliations:** 1 Department of Neurogenetics, Max Planck Institute of Experimental Medicine, Göttingen, Germany; 2 International Max Planck Research School Neurosciences, Göttingen Graduate School for Neurosciences and Molecular Biosciences, Göttingen, Germany; 3 Auditory Systems Physiology Group, InnerEarLab, Department of Otolaryngology, University Medical Center, Göttingen, Germany; 4 Department of Molecular Neurobiology, Max Planck Institute of Experimental Medicine, Göttingen, Germany; 5 Holon Institute of Technology, Holon, Israel; New York University, United States of America

## Abstract

Detecting regular patterns in the environment, a process known as statistical learning, is essential for survival. Neuronal adaptation is a key mechanism in the detection of patterns that are continuously repeated across short (seconds to minutes) temporal windows. Here, we found in mice that a subcortical structure in the auditory midbrain was sensitive to patterns that were repeated discontinuously, in a temporally sparse manner, across windows of minutes to hours. Using a combination of behavioral, electrophysiological, and molecular approaches, we found changes in neuronal response gain that varied in mechanism with the degree of sound predictability and resulted in changes in frequency coding. Analysis of population activity (structural tuning) revealed an increase in frequency classification accuracy in the context of increased overlap in responses across frequencies. The increase in accuracy and overlap was paralleled at the behavioral level in an increase in generalization in the absence of diminished discrimination. Gain modulation was accompanied by changes in gene and protein expression, indicative of long-term plasticity. Physiological changes were largely independent of corticofugal feedback, and no changes were seen in upstream cochlear nucleus responses, suggesting a key role of the auditory midbrain in sensory gating. Subsequent behavior demonstrated learning of predictable and random patterns and their importance in auditory conditioning. Using longer timescales than previously explored, the combined data show that the auditory midbrain codes statistical learning of temporally sparse patterns, a process that is critical for the detection of relevant stimuli in the constant soundscape that the animal navigates through.

## Author summary

Some things are learned simply because they are there and not because they are relevant at that moment in time. This is particularly true of surrounding sounds, which we process automatically and continuously, detecting their repetitive patterns or singularities. Learning about rewards and punishment is typically attributed to cortical structures in the brain and known to occur over long time windows. Learning of surrounding regularities, on the other hand, is attributed to subcortical structures and has been shown to occur in seconds. The brain can, however, also detect the regularity in sounds that are discontinuously repeated across intervals of minutes and hours. For example, we learn to identify people by the sound of their steps through an unconscious process involving repeated but isolated exposures to the coappearance of sound and person. Here, we show that a subcortical structure, the auditory midbrain, can code such temporally spread regularities. Neurons in the auditory midbrain changed their response pattern in mice that heard a fixed tone whenever they went into one room in the environment they lived in. Learning of temporally spread sound patterns can, therefore, occur in subcortical structures.

## Introduction

As we interact with the environment, our brain is constantly detecting patterns—i.e., regularities—in the sensory world. This capacity allows us to recognize surrounding stimuli and make predictions necessary for survival. Patterns in the sensory input are extracted through a process known as statistical learning [1]. Regularities in the continuous sensory input that fit relatively short windows, in the order of seconds to tens of seconds, can be encoded through neuronal adaptation of response gain in both subcortical and cortical structures [2–4]. However, little is known about the circuits that code patterns that are temporally sparse, i.e., when the regularity is repeated discontinuously across time windows of minutes and hours. Statistical learning of sparse patterns is important for grammatical learning or musical sensitivity in humans [5,6], both of which are achieved through exposures that occur across days to years. This type of learning is likely to involve long-term plasticity mechanisms, different from neuronal adaptation.

Changes in neuronal response gain that reflect fast adaptation are ubiquitous in the auditory cortex (AC) [2,7,8] but can also be found in the inferior colliculus, a subcortical midbrain structure that is the first convergence station in the auditory circuit [9]. For example, stimulus probability selectivity [3,4,10,11], as well as some forms of response selectivity to natural sounds [12–14], is observed in some divisions of the inferior colliculus [4]. Correlations between inferior colliculus activity and temporal patterns, such as speech or rhythmic tapping, have also been described in humans [11,12]. We hypothesized that neuronal correlates of statistical learning of temporally sparse patterns can also be found in the inferior colliculus.

The context can be a strong predictor of the soundscape. In real life, as animals move through the environment, they can reencounter the same context and its characteristic sounds in temporally spread bouts. Here, in order to understand the neuronal coding of temporally sparse patterns in the sensory input, we used context–sound associations as stimuli. Thus, we set out to specifically test (1) whether mice can detect temporally sparse context–sound associations and (2) whether this detection triggers changes in the response patterns of neurons in the inferior colliculus.

To recreate a natural environment while maintaining control over the experimental variables, we used the Audiobox—a socially, acoustically, and behaviorally enriched environment in which mice lived in groups for up to 2 weeks [15]. Mice were exposed to sounds that were associated with the context, with different degrees of predictability. The consequence of this exposure was assessed at the behavioral, electrophysiological, and molecular levels. First, we measured the effect that temporally sparse sound exposure had on the response gain of collicular neurons by simultaneously measuring evoked responses across different frequency bands. We subsequently assessed the effect these changes had on frequency coding and discrimination before testing how physiological changes in sensory gating paralleled behavioral generalization measures. We then confirmed that plasticity-associated changes in gene and protein expression had taken place. Since conditioning-triggered midbrain plasticity can depend on corticofugal input [16], we tested the dependence of the observed changes on cortical feedback. Finally, to ascertain the origin of changes in the activity of inferior colliculus neurons, we assessed the effect that sound exposure had on upstream and downstream structures.

## Results

**Fig 1:**
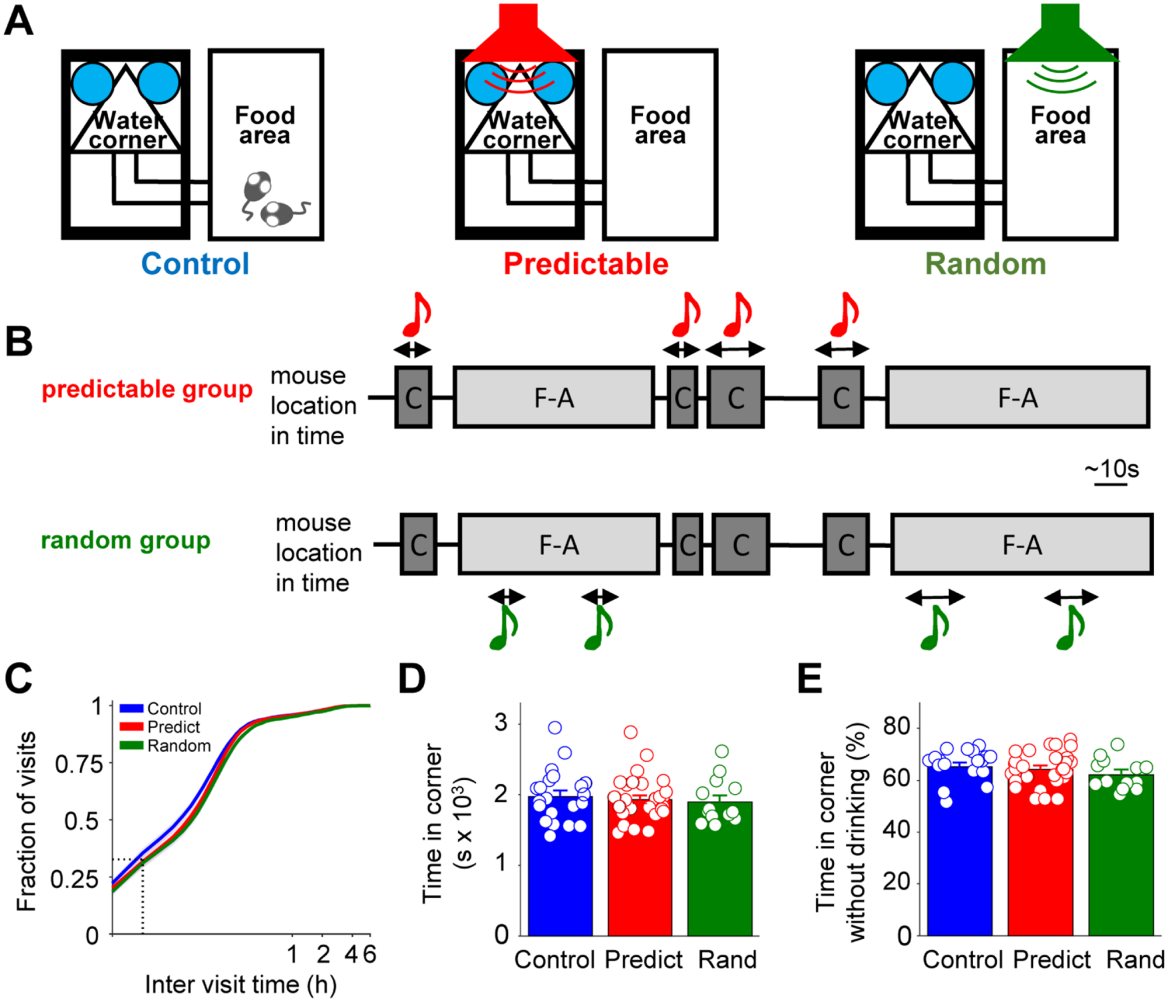
Sound exposure does not affect ongoing behavior in the Audiobox. (A) Schematic representation of the Audiobox and exposure protocols. Water was available in the water corner and food in the food area. Sound exposure took place in the water corner in every visit (predictable group, center), at random times in the food area (random group, right), or not at all (control group, left). (B) Schematic representation of the temporal association between visits to the water corner (“C”) and visits to the food area (“F-A”) and the sound in the predictable (top) and random (bottom) groups. (C) Cumulative distribution of the intervisit time interval to the water corner area. The dotted lines indicate the fraction of visits within 1 minute of intervisit time. (D) Mean daily time spent in the water corner area was similar between groups (ANOVA, F_2,60_ = 0.24, *p* = 0.78). For B-D: control *n* = 21; predictable *n* = 29; random *n* = 13. All animals used for electrophysiology were included here. (E) Mean daily percentage of time spent in the water corner area without drinking was similar between groups (ANOVA, F_2,60_ = 0.98, *p* = 0.38). Error bars represent SEM. Numerical data for this figure can be found in S1 Data.

We first established a naturalistic behavioral setting to study the learning of sparse context–sound associations. All mice used in these series of experiments were exposed to sounds in the Audiobox (Fig 1A), where mice lived in groups of 8–10 individuals for 6–12 days. Food and water could be found ad libitum at opposite ends of the apparatus. Water was available in a specialized corner separated from the food area by a corridor. We designed an experimental paradigm of auditory statistical learning with different degrees of predictability of sound exposure. Three groups of mice were tested, a “predictable” group, a “random” group, and a control group. The mice in the predictable group heard a fixed pure tone of 16 kHz every time they visited the water corner (Fig 1A, center). This sound was presented in pips for the duration of the visit, independently of whether the mice nose-poked and drank or not (Fig 1B, top). The sound was fully predictable, for it was triggered by the animal itself. In the random group, mice heard the same pure tone randomly in the food area (Fig 1A, right). This tone was triggered in a yoke control design by a mouse living in a different Audiobox whenever she entered the water corner. Thus, sound presentation had the same temporal pattern as in the predictable group, both in terms of time of appearance (mainly in the dark cycle) and typical duration (corresponding to water corner visits’ length), but was not predictable (Fig 1B, bottom). A control group of mice lived in the Audiobox for the same length of time as mice in the two other groups. They heard the background sounds intrinsic to the environment and their own movements, such as opening of the sliding doors upon nose-poke; but, unlike mice in the predictable and random groups, they heard no sounds that came out of a speaker (Fig 1A, left). Sound exposure was temporally sparse, with bouts of sound presentation typically separated by over 5 minutes (Fig 1C) and lasting less than 15 seconds (S1A Fig). These three different modes of sound exposure had no effect on the animal’s behavior (Fig 1D and 1E), consistent with the fact that the sounds did not trigger explicit reward or punishment. The daily time spent in the water corner was comparable across groups (Fig 1D). In all groups, more than 60% of this time was spent without nose-poking for water (Fig 1E), and over 25% of all visits to the corner were not accompanied by a nose-poke (S1B Fig).

### Predictable sound exposure generates sound–context associations

We did not find changes in the animal’s behavior during sound exposure that could indicate learning of the context–sound association. In order to ascertain whether statistical learning had occurred, we tested the effect that the different exposure patterns had on subsequent conditioned frequency discrimination. For that purpose, we used latent inhibition (LI) [17,18]. LI is the effect by which exposure to a neutral, nonreinforced stimulus delays learning of a subsequent association between this stimulus and an aversive outcome. We have shown before [15] that the mere exposure to a sound in the corner elicits LI in the Audiobox when the sound is subsequently conditioned in the same place, indicating that the presence of the sound in the corner was learned. We now probed the conditions under which LI is observed by comparing the effect of predictable and random sound exposure. Following the predictable or random sound exposure phases (16 kHz; S1C Fig and Methods), all mice were conditioned to 16 kHz sound in some visits to the water corner, such that a nose-poke during conditioned visits would trigger the delivery of an aversive air puff (S1D Fig). Mice needed to discriminate between safe visits and conditioned visits and refrain from nose-poking during the latter. On the first day of conditioning, the control (never exposed to 16 kHz) and random (exposed to 16 kHz outside the corner) groups showed successful avoidance when 16 kHz was present in the corner and good discrimination, as reflected in d′ values above 1 (S1E Fig). The predictable group, on the other hand, had d′ values significantly below the other groups (S1E Fig), indicating the failure to avoid nose-poking when 16 kHz was present, i.e., the occurrence of LI. This indicates that mice had learned the association between the safe 16 kHz tone and the corner during the exposure phase. Note that random sound exposure in the food area had a mild effect on the levels of avoidance in the corner during conditioning (S1E and S1F Fig, green triangles), and mice never reached the level of performance of the control group, suggesting that both forms of sound exposure influenced subsequent avoidance during conditioned visits, albeit with weaker effects when random. In summary, all three groups behaved identically during the exposure phase but showed three different patterns of behavior during subsequent conditioning of the 16 kHz sound in the corner. Thus, learning of the association between the predictable sound and the context where it was heard (the water corner) did occur even though it had no effect on behavioral measures during the exposure itself. We conclude that the exposure protocol constitutes a successful model of temporally sparse statistical learning.

### Sound exposure increases evoked responses in the inferior colliculus

**Fig 2:**
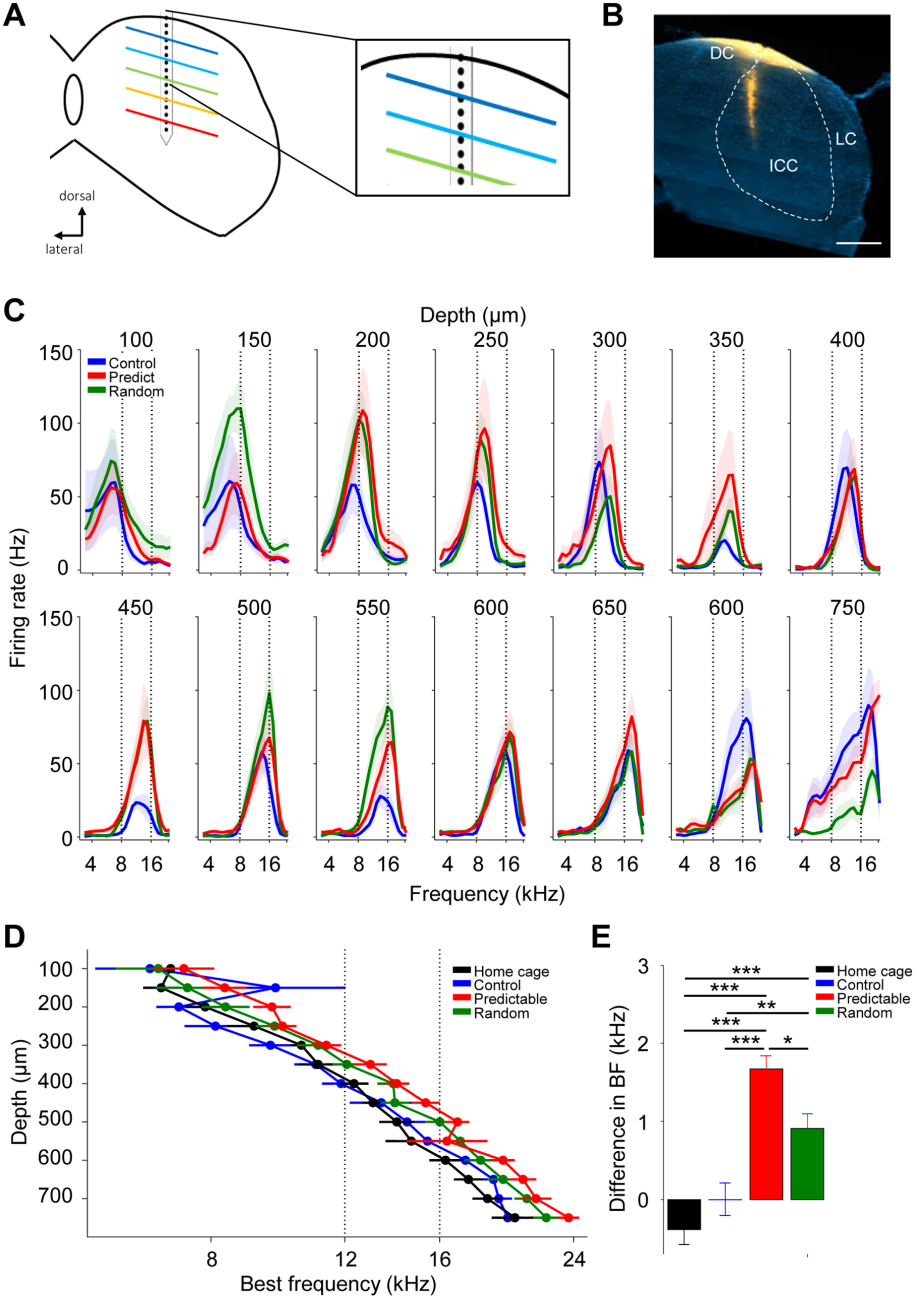
Sound exposure results in increases in response gain in the inferior colliculus. (A) Left, schematic representation of the recording approach in the inferior colliculus using linear multielectrode array. Inset: Schematic representation of positioning of most superficial recording site, aligned with dura. (B) Right, representative dorsoventral electrode penetration track (DiI) through dorsal cortex and central nucleus. “DC”: dorsal cortex; “ICC”: central nucleus; “LC”: lateral cortex. Scale bar 500 μm. (C) Mean tuning curves of simultaneously recorded evoked responses (70 dB) for different depths in the inferior colliculus (linear mixed effects model; group × depth interaction F_2,8412_ = 4.21, *p* < 0.05). Animals and recording sites: control *n* = 10 and 98; predictable *n* = 14 and 162; and random *n* = 7 and 91. (D) Mean collicular BF for different depths in the inferior colliculus (ANOVA, group F_3,334_ = 10.89; *p* < 0.0001). Animals and recording sites for D-E: home cage *n* = 6 and 72; control *n* = 10 and 98; predictable *n* = 14 and 162; and random *n* = 7 and 91. (E) Mean BF difference across the tonotopic axis with respect to the mean BF of control group (ANOVA, group F_3,386_ = 9.97, *p* < 0.0001. Corrected pair comparisons: *p < 0.05, **p < 0.01, ***p < 0.001). Error bars represent SEM. Numerical data for this figure can be found in S1 Data. BF, best frequency; DiI, 1,1'-dioactedecyl-3,3,3,3'-tethramethyl indocarbocyanide.

The inferior colliculus is an auditory subcortical station on which diverse sensory information converges [9]. It has been shown to be sensitive to short-term statistical learning through neuronal adaptation. We now investigated whether statistical learning of temporally sparse patterns could affect the coding properties of the inferior colliculus. We acutely recorded from the inferior colliculus of anesthetized animals exposed to predictable or random 16 kHz for 6–12 days (Fig 1A and 1B). We recorded multiunit activity from well-separated spikes (S2A Fig) using linear multielectrode arrays (16 sites, 50 μm apart) inserted dorsoventrally along the collicular tonotopic axis (Fig 2A and 2B). The first electrode was on the dura, and the second electrode rarely gave reliable responses. We therefore characterized auditory-evoked responses to different tone frequency–intensity combinations simultaneously in the remaining 14 depths (100–750 μm, see Methods). Depths of 100 and 150 μm were considered to be putative dorsal cortex based on different response patterns [19,20], and the remaining depths, the central nucleus. All experimental groups showed a dorsoventral axis of tonotopic organization in the inferior colliculus such that progressively higher frequencies elicited responses progressively deeper (Fig 2C; representative example raster plots in S2B–S2D Fig), in agreement with previous studies [21,22]. Tuning was quantified using spikes evoked at 70 dB SPL (behavioral mean exposure intensity was 68 dB) by stimuli of 30 ms length (see Methods). An increase in response gain was evident in the tuning curves of predictable animals with respect to control animals at multiple depths along the tonotopic axis of the inferior colliculus (Fig 2C). The predictable group had homogenously high levels of activity across all depths (see Fig 2C, red, for mean). The random group had high activity localized to the putative dorsal cortex (<200 μm depth) and to depths with best frequencies (BFs; the frequency that elicits the strongest response in a given location) around 16 kHz (500–550 μm: Fig 2C and S2E Fig, green). This pattern of responses in the predictable and random groups was confirmed by quantification of peak firing rates in depth zones (S3A Fig). The overall mean peak of firing rate of the control group was similar to age-matched animals reared under standard conditions (home cage group) but significantly smaller than the predictable group (S3B Fig). Thus, sound exposure, whether predictable or random, generated an increase in collicular evoked activity compared to control animals. While in the random group, the increase was localized to depths with good responses at and near 16 kHz; in the predictable group, it was homogeneously distributed. The effect was not dependent on the frequency of the exposed tone, since mice in a predictable group exposed to frequencies other than 16 kHz also showed an increase in response gain (S3C Fig for group exposed to 8 kHz). The effect was not dependent on the number of exposure days (6–12 days) in the Audiobox (S3D and S3E Fig). When individual tuning curves were aligned by BF rather than depth, the overall increase in excitability in the predictable group remained (S3F Fig).

### Sound exposure leads to a global suprathreshold shift in BF

Experience-dependent plasticity, such as auditory conditioning, can induce transient shifts in the BF of collicular neurons [23–25]. Indeed, we noticed that the peaks of the tuning curves of the predictable group were shifted in multiple depths (Fig 2C, e.g., 300–500 μm) compared to the control group. Unlike what has been reported before as a result of conditioning, the shift in BFs that resulted from sound exposure was not toward the conditioned frequency but toward higher frequencies, even in regions with BFs of 16 kHz or above. The average BFs were consistently higher in the predictable and, to a lesser extent, the random group than in the control and home cage groups (Fig 2D). Further quantification of the mean difference in BF across depth with respect to the control group confirmed this effect (Fig 2E).

The BF shift was independent of the frequency of the sound played in the water corner area. We measured the BFs in animals that were exposed under identical conditions to frequencies different from 16 kHz (either 8 kHz, 13 kHz, or a combination of 8 and 13 kHz). Except for the group exposed to 8 kHz alone, which did not show a reliable shift in BF with respect to controls (but note shifts in this group at specific depths, S3C Fig), shifts were similar in magnitude to those observed in mice exposed to 16 kHz (S4A Fig; see Methods). Interestingly, average BF at threshold intensities was similar between groups (S4B Fig), indicating that the shift is in suprathreshold tuning rather than a real change in tonotopy. Care was taken during the probe insertion to ensure consistency in the location and depth of the electrodes (see Methods), and small variations from animal to animal cannot explain the systematic group differences. Additionally, simultaneous recordings along the rostrocaudal axis of predictable and control animals (S4C Fig; see Methods) revealed that the upward shift was present throughout the dorsoventral axis in the rostral and caudal portions of the inferior colliculus. In summary, there was a homogenous, frequency-unspecific, and suprathreshold shift in tuning in both exposed groups. The shift was significantly stronger in the predictable group and, unlike previously described for conditioning paradigms [23–25], the shift was not toward the exposed frequency but upward along the tonotopic axis.

### Predictable and random sound exposure increases response gain through different mechanisms

**Fig 3:**
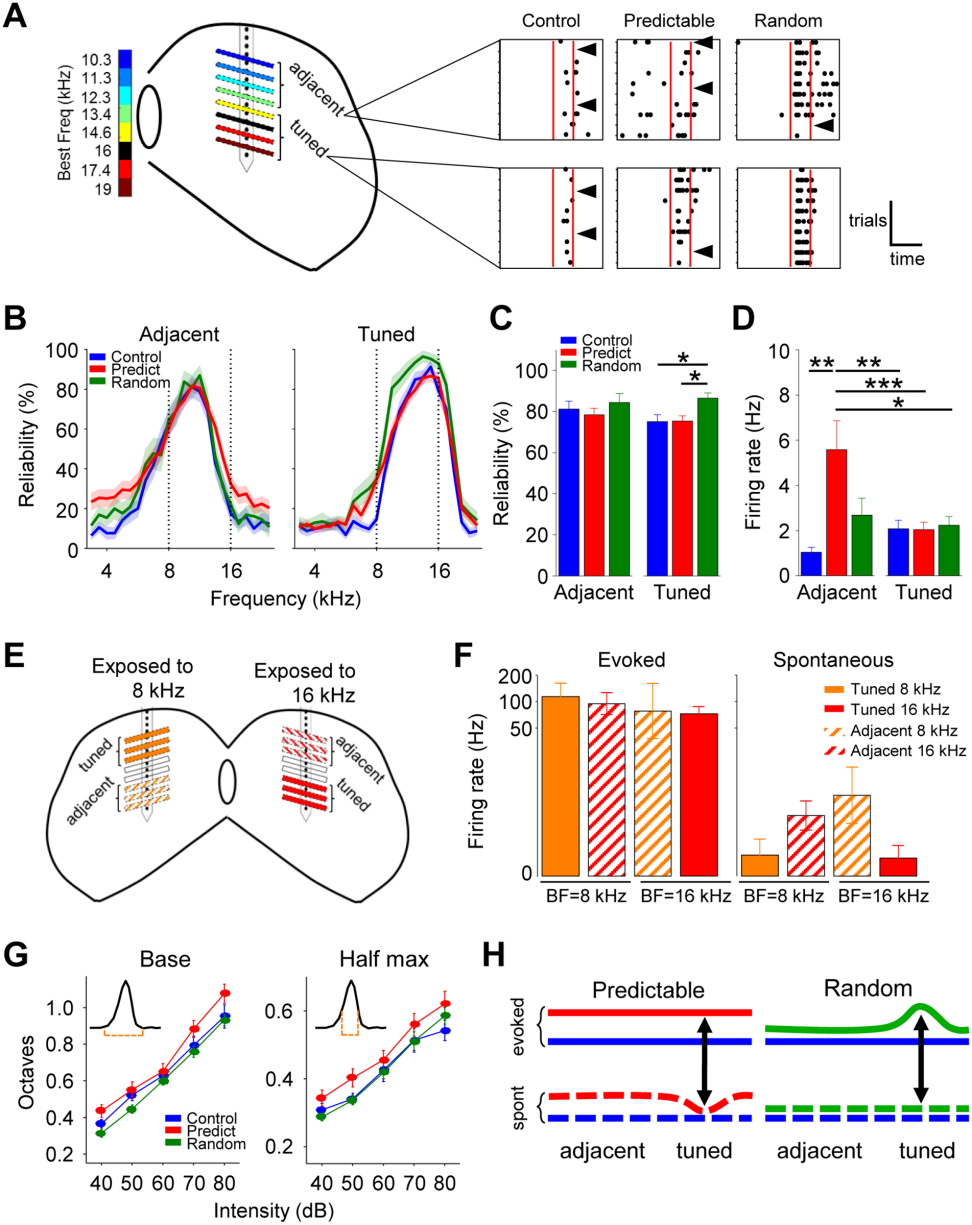
Predictable and random sound exposure differentially modulate response gain in the inferior colliculus. (A) Left, schematic representation of the adjacent and tuned regions. Right, example responses to the peak of the tuning in adjacent (top) and tuned (bottom) regions for each group. Dots are spikes. Vertical axis is trials. Red lines indicate stimulus duration. Black arrows indicate trials without evoked spikes. (B) Left, mean response reliability (trials with at least 1 evoked spike, 80 ms from stimulus onset, 70 dB) as a function of frequency for the adjacent area (ANOVA, group F_2,2784_ = 20.18, *p* < 0.0001. Corrected pair comparisons: *p* < 0.0001 control versus predictable; *p* = 0.081 control versus random; *p* < 0.01 predictable versus random). For B-D adjacent: animals and recording sites: control *n* = 10 and 34; predictable *n* = 14 and 60; and random *n* = 7 and 26. Right, same as left for the tuned area (ANOVA, group F_2,3336_ = 32.29, *p* < 0.0001. Corrected pair comparisons: *p* < 0.01 control versus predictable; *p* < 0.0001 control versus random; *p* < 0.0001 predictable versus random). For B-D tuned: animals and recording sites: control *n* = 10 and 42; predictable *n* = 14 and 68; and random *n* = 7 and 34. (C) Mean response reliability to the respective BF ± 0.25 octaves for the adjacent (left, ANOVA, F_2,112_ = 0.66, *p* = 0.51) and tuned (right, ANOVA, F_2,136_ = 4.13, *p* = 0.018. Corrected pair comparisons: *p < 0.05) regions. (D) Mean spontaneous activity for adjacent (left) and tuned regions (right; ANOVA, F_5,255_ = 4.71, *p* < 0.001. Corrected pair comparisons: *p < 0.05; **p < 0.01; ***p < 0.001). (E) Schematic representation of adjacent and tuned regions in the comparison between two predictable groups, one exposed to 8 kHz and the other exposed to 16 kHz. (F) Left, mean firing rate evoked by the BF in depths with a BF of 8 or 16 kHz, for animals exposed to 8 kHz or 16 kHz. Right, same as left for the spontaneous activity (exposed to 16 kHz *n* = 9; exposed to 8 kHz *n* = 3). (G) Mean bandwidth as a function of sound intensity measured at the base (top) or at the half-maximum (bottom) of the tuning curve (left, base ANOVA, group F_2,674_ = 7.85, *p* < 0.001. Corrected pair comparisons: *p* < 0.05 control versus predictable; *p* < 0.001 predictable versus random; right, half-maximum: ANOVA, group F_2,674_ = 4.9, *p* < 0.01. Corrected pair comparisons: *p* < 0.05 control versus predictable; *p* < 0.05 predictable versus random). Animals and recording sites: control *n* = 7 and 35–43; predictable *n* = 8 and 61–72; random *n* = 7 and 30–35 recording sites. Error bars represent SEM. (H) Model of the differential plasticity produced in the inferior colliculus upon predictable (left) or random (right) sound exposure. Left, in the predictable group, the increase in response gain was homogenous (continuous red line) and, excepting the tuned area, also affected spontaneous activity (dotted line). Right, in the random group, the increase in response gain was the result of increased local reliability in the tuned area without affecting spontaneous activity. Numerical data for this figure can be found in S1 Data. BF, best frequency.

Experience-dependent plasticity often results in changes in response gain [26,27], which can take the shape of changes in response reliability, spontaneous activity, signal-to-noise ratio (SNR), and tuning bandwidth [28,29]. To evaluate which of these variables was responsible for the increase in response gain in the predictable and random groups in a frequency-specific manner, we divided recording sites in 2 equally sized regions: one of sites with a BF tuned around 16 kHz (14–19 kHz, “tuned” hereafter; Fig 3A) and another with sites tuned to 10–13 kHz (“adjacent” hereafter; Fig 3A). We first measured whether the increase in gain was the result of an increase in firing rate alone or also in the reliability of evoked responses (defined as the percentage of trials with at least 1 spike during the evoked period, 0–80 ms from stimulus onset; example in Fig 3A, right). In both the tuned and adjacent regions, response reliability was stronger around the local BF and decreased toward the edges of the frequency range, mirroring tuning (Fig 3B). In the tuned region (Fig 3B, right), the reliability of the evoked responses was significantly higher in the random group compared to the other groups, as quantified for the peak of tuning (Fig 3C, right; see example in Fig 3A, right). On the other hand, spontaneous activity was similar across groups in the tuned region but higher for the predictable group in the adjacent region (Fig 3D; see example in Fig 3A, right).

If only adjacent regions showed an increase in spontaneous activity, mice exposed to a tone in the low frequency range (8 kHz) would show a converse pattern: an increase in spontaneous activity in the region that we now call tuned (Fig 3E). Indeed, when mice were exposed to 8 instead of 16 kHz, we found that the spontaneous activity was increased in the area with BFs near 16 kHz and comparable in the regions with BFs near 8 kHz (Fig 3F). The region-specific increase in spontaneous activity had a direct effect on the SNR (evoked/spontaneous firing rate), which was significantly smaller in the adjacent region compared to the tuned region in the predictable group (S5A Fig). We conclude that the SNR increased in the area that responds to the exposed tone, independently of its frequency, compared to the flanking regions.

Finally, tuning bandwidth was increased in the predictable group with respect to both control and random groups. The effect was observed at both the base and half-maximum of the tuning curve (Fig 3G, left and right respectively). Changes in gain were not the result of changes in overall excitability, since intensity thresholds were similar (35 dB) in all groups (S5B Fig). Additionally, we quantified response latency (see Methods), which is known to decrease with the efficiency of the stimulus [30]. In the predictable group, latencies were similar in both regions compared to the control group (S5C Fig). In the random group, latencies were lower than the control group in the adjacent region and lower than the predictable group in the tuned region (S5C Fig).

To conclude, the increase in response gain observed in the predictable and random groups resulted from different mechanisms (Fig 3H). In the predictable group, the increase in response gain was frequency unspecific and affected the evoked and the spontaneous activity, as well as the tuning bandwidths. Moreover, spontaneous activity was reduced in the tuned region, resulting in a local increase in SNR. In the random group, the increase in evoked activity was centered around the exposure frequency and was, at least in part, the result of increased reliability without affecting either spontaneous activity or tuning bandwidth.

### Increase in response gain affects population activity, reflected in the structural tuning

**Fig 4:**
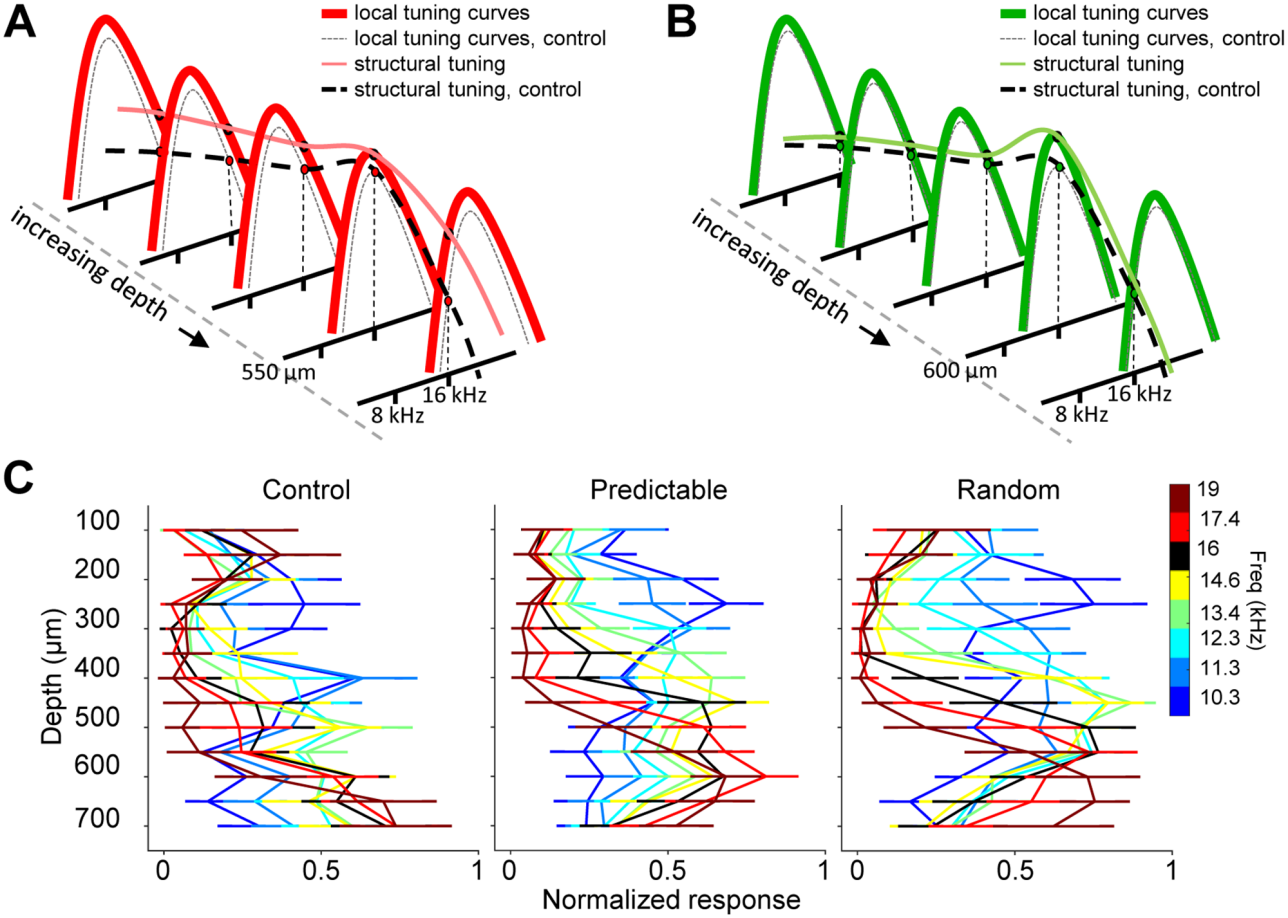
Predictable sound exposure modifies structural tuning. (A) Scheme of the local tuning curves and the structural tuning along the collicular tonotopic axis for the predictable (red) and the control (black) groups. (B) Same as A for the random (green) and the control (black) groups. (C) Mean normalized structural tuning, evoked response across depths, for a subset of frequencies (ANOVA, group × depth × frequency interaction F_168,1869_ = 2.34, *p* < 0.0001). Animals and recording sites: control *n* = 10 and 98; predictable *n* = 14 and 162; random *n* = 7 and 91. Numerical data for this figure found in S1 Data.

Auditory input evokes responses throughout the tonotopic map. This is reflected in neither peri-stimulus time histogram (PSTH) nor tuning curves, both of which represent local responses. Since we recorded simultaneously from 14 locations along 700 μm of the inferior colliculus, we were able to quantify the simultaneous response to a given frequency along the collicular tonotopic axis. We will refer to this response as structural tuning (Fig 4A and 4B). Unspecific increases in bandwidth, such as that observed in the predictable group, would have the effect of increasing the response gain to a given frequency tone throughout the tonotopic map (Fig 4A, light red versus dashed structural tuning). Increases in reliability that are not accompanied by changes in tuning bandwidth, such as that observed in the random group, would have the effect of increasing a structural tuning curve’s gain at a local depth without much change elsewhere (Fig 4B, light green versus dashed structural tuning curves). Indeed, sound exposure affected structural tuning curves of different frequencies for the predictable and random groups, which were more distinct across frequencies compared to those of control animals (Fig 4C). The effect this has on coding will be assessed below.

### Differential increase in response gain results in differential frequency coding and discrimination

**Fig 5:**
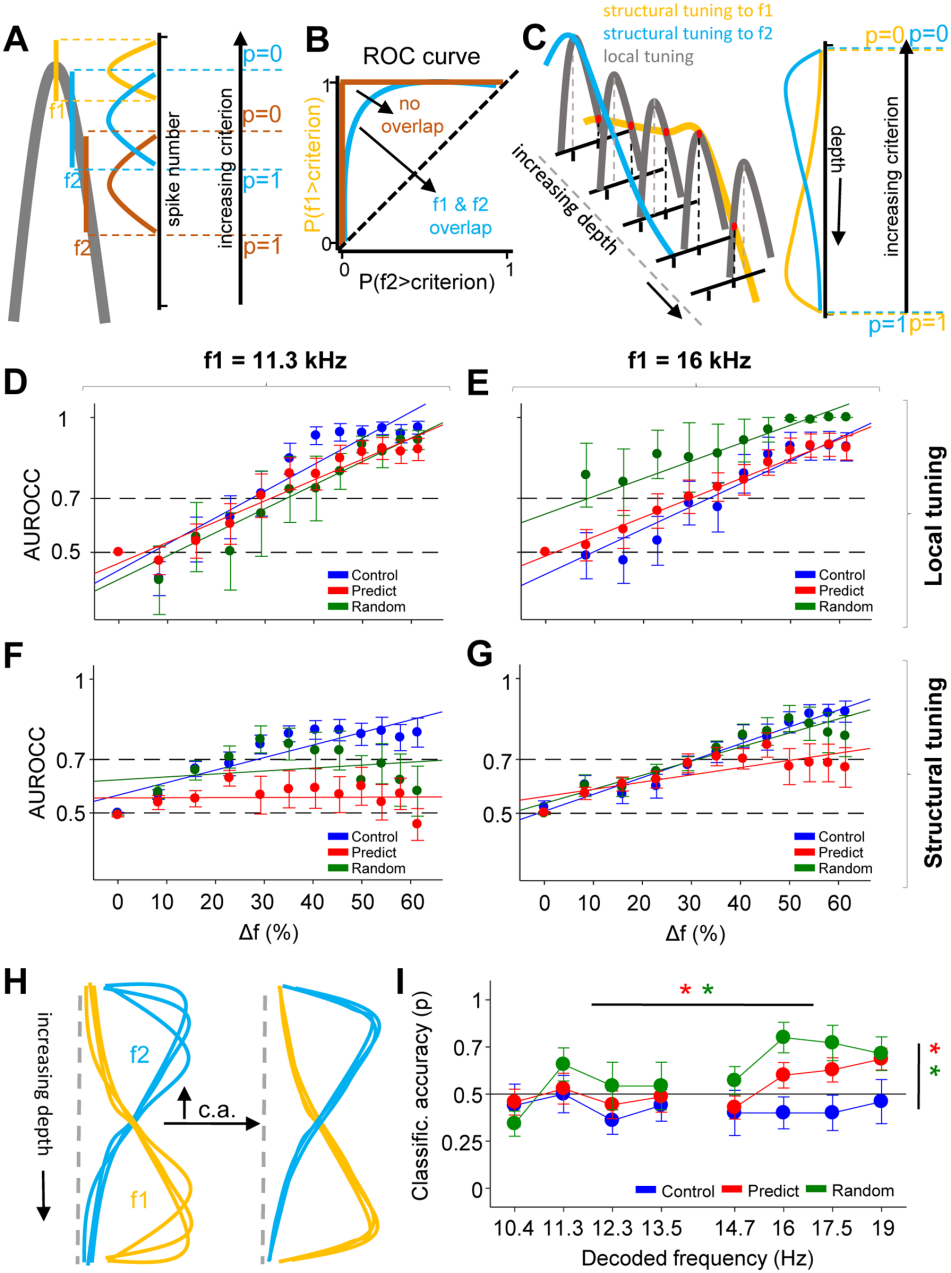
Predictable sound exposure leads to increased overlap in structural tuning and better classification accuracy. (A) Scheme that illustrates the computing of the ROC curves using the local tuning curves, in which f1 is the BF. (B) Scheme that illustrates the example of a ROC curve. (C) Same as D but for the structural tuning. (D) AUROCC calculated from the tuning curves with BF of 16 kHz ± 1.1% (tuned region) across groups and ΔF. Each point is the comparison between an f1 of 16 kHz and an f2 of a frequency separated by a given ΔF. (ANOVA, group F_2,324_ = 11.78, *p* < 0.0001, ΔF F_11,324_ = 14.49, *p* < 0.0001). Animals: control *n* = 10; predictable *n* = 14; random *n* = 7. Corrected pair comparisons: *p* < 0.0001 random versus control, *p* < 0.0001 random versus predictable. (E) Same as D for tuning curves with BF of 11.31 kHz 1.1% (adjacent region). Here, f1 was 11.3 kHz throughout. (ANOVA, group F_2,552_ = 8.17, *p* < 0.0001, ΔF F_11,552_ = 17.08, *p* < 0.0001). Animals: control *n* = 10; predictable *n* = 14; random *n* = 7. Corrected pair comparisons: *p* = 0.019 random versus control, *p* = 0.0003 predictable versus control. (F) AUROCC calculated from the structural tuning curves with BF of 16 kHz ± 1.1% (tuned region) across groups and ΔF. Each point is the comparison between the mean of responses to f1 of 16 kHz and individual responses to f2. (ANOVA, group F_2,336_ = 9.37, *p* = 0.0001, ΔF F_11,336_ = 12.1, *p* < 0.0001). Animals: control *n* = 10; predictable *n* = 14; random *n* = 7. Corrected pair comparisons: *p* = 0.0003 predictable versus control, *p* = 0.0053 predictable versus random. (G) Same as in F, using an f1 of 11.3 kHz. (ANOVA, group F_2,335_ = 33.34, *p* < 0.0001, ΔF F_11,335_ = 3.94, *p* < 0.0001). Animals: control *n* = 10; predictable *n* = 14; random *n* = 7. Corrected pair comparisons: *p* < 0.0001 predictable versus control, *p* = 0.023 random versus control, *p* = 0.0001 predictable versus random. (H) Scheme illustrating the relationship between ROC and classification accuracy (labeled “c.a.”). Upward arrow equals increased classification accuracy. (I) Mean classification accuracy probability for frequencies in the adjacent (BF of 10–13 kHz) and tuned (BF of 16–19 kHz) regions. Error bars represent SEM. (ANOVA, group F_2,247_ = 7.37, *p* = 0.0008, region F_1,247_ = 5.78, *p* = 0.017, frequency F_3,247_ = 2.49, *p* = 0.061. In the tuned region: ANOVA, group F_2,123_ = 9.44, *p* = 0.000, corrected pair comparisons: *p* = 0.011 predictable versus control, *p* = 0.0001 random versus control. For control group: ANOVA, region F_1,79_ = 0.07, *p* = 0.78. For predictable group: ANOVA, region F_1,111_ = 4.04, *p* = 0.046. For random group: ANOVA, region F_1,55_ = 7.01, *p* = 0.010). Animals: control *n* = 10; predictable *n* = 14; random *n* = 7. Numerical data for this figure found in S1 Data. AUROCC, area under the ROC curve; BF, best frequency; ROC, receiver operating characteristic.

We assessed how different changes in response gain across groups both locally (region specific, tuning curves) and globally (structural tuning) affected frequency coding and discrimination. We measured between-frequency discrimination and within-frequency response consistency using receiver operating characteristic (ROC) curve analysis and classification accuracy measures, respectively. ROC analysis is used to assess discriminability between two stimuli [31] by comparing the cumulative probability distributions of responses to these stimuli for different discrimination criteria (Fig 5A and 5B). For the local tuning, we used individual tuning curves with a BF of 11.3 kHz ± 1.1% (adjacent region) or 16 kHz ± 1.1% (tuned region) and generated ROC curves for comparison between the BF and the to-be-compared frequency (f1 and f2 in Fig 5A). We then used the area under the ROC curve (AUROCC, Fig 5B) as the index of discriminability. ROC curves obtained from tuning curves in the adjacent region were not different between predictable and random groups (Fig 5D). In the tuned region, however, the random group showed better discrimination (larger AUROCC) for all ΔFs than both the control and predictable groups, who do not differ between them (Fig 5E). This region-specific increase in discriminability in the random group parallels the region-specific increase in both gain and reliability in this group, in the absence of a change in bandwidth. In the predictable group, there was no change in discriminability in either region, which is consistent with the region-unspecific increase in both gain and bandwidth (Fig 5D and 5E). This consistency derives from the fact that ROC curves are not sensitive to changes in response size, only to changes in distributions, and these are not necessarily changed when gain and bandwidth increase together.

We then performed the same analysis for the structural tuning. This was performed for individual responses to a given frequency compared to the mean response (across trials) to 11.3 kHz (Fig 5F) and 16 kHz (Fig 5G). Here, the predictable group shows less discriminability between frequency pairs (Fig 5F, in which f1 = 11.3 kHz, and Fig 5G, in which f1 = 16 kHz) than both the random and control groups. This decrease in discriminability in the predictable group is consistent with the increase in bandwidth and the concomitant increase in activity throughout the structural tuning curve (see Fig 4A), which ultimately changes response distribution across the tonotopic axis and increases overlap between structural tuning curves.

To a certain extent, ROC analysis reflects the variability in the response to each of the stimuli compared. Yet this is not true for the structural tuning ROC curves, because their wide response distributions (responses across all depths) and their asymmetrical shapes (Fig 5C) increase the level of overlap between the distribution curves without reflecting the trial-to-trial variability at the peak of the distribution (Fig 5H). Trial-to-trial response consistency can be measured using classification accuracy probabilities. We used structural tuning curves to train a classifier [32,33] to predict the played frequency (see Methods). The probability of predicting a given frequency correctly was significantly higher in both predictable and random groups with respect to control. In both groups, accuracy was higher in the tuned versus the adjacent region (Fig 5I).

Overall, the data suggest that statistical learning is accompanied by changes in neuronal coding in the inferior colliculus that affect frequency discrimination and response classification accuracy.

### Predictable sound exposure decreases behavioral spontaneous frequency discrimination acuity

**Fig 6:**
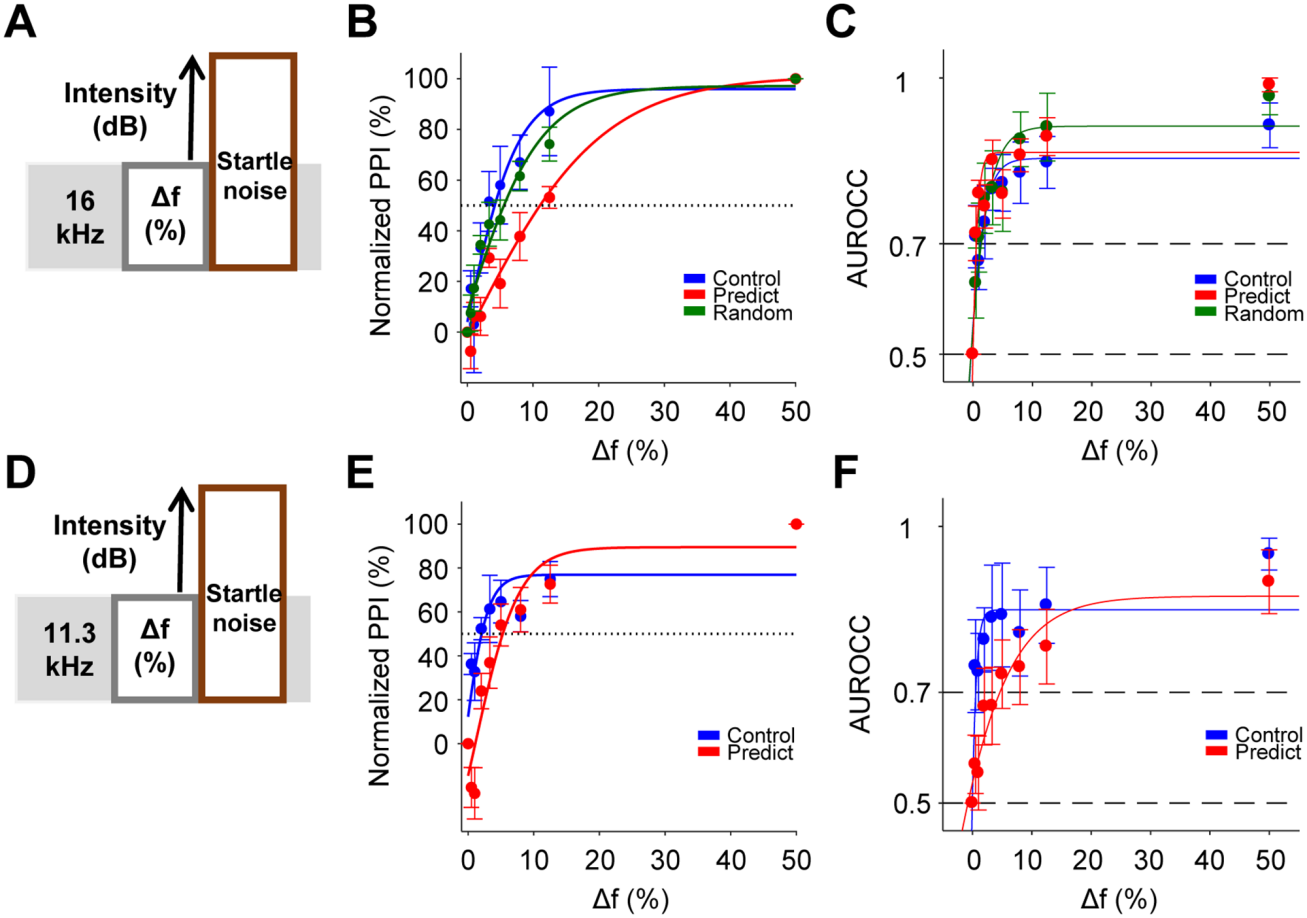
Predictable sound exposure increases behavioral spontaneous frequency generalization but reduces trial-to-trial variability. (A) Scheme of a single PPI trial: startle noise was preceded by a prepulse tone with a Δf of between 0% and 50% below the background tone of 16 kHz. (B) Normalized PPI as a function of frequency change between the prepulse and the background tone of 16 kHz for control, predictable, and random groups. Continuous line indicates a fitted logistic function (ANOVA, group F_2,189_ = 13.59, *p* < 0.01; corrected pair comparisons: *p* < 0.001 predictable versus control and *p* < 0.01 predictable versus random). Dash line: discrimination threshold. Control *n* = 7; predictable *n* = 8; random *n* = 9. (C) Mean AUROCCs calculated from the behavioral data in B. Continuous line indicates a fitted logistic function (ANOVA, group F_2,189_ = 1.3, *p* = 0.27. Control *n* = 7; predictable *n* = 8; random *n* = 9). (D) Scheme of a single PPI trial with a tone background of 11.3 kHz. (E) Normalized PPI as a function of frequency change between the prepulse and the background tone of 11.3 kHz for control and predictable groups. Continuous line indicates a fitted logistic function (ANOVA, group F_2,81_ = 18.92, *p* < 0.0001; group × frequency interaction F_2,81_ = 3.06, *p* < 0.01). Control *n* = 4; predictable *n* = 7. (F) Mean AUROCCs calculated from the behavioral data in E. Continuous line indicates a fitted logistic function (ANOVA, group F_2,81_ = 10.36, *p* = 0.0019). Control *n* = 4; predictable *n* = 7. Error bars represent SEM. Numerical data for this figure found in S1 Data. AUROCC, area under ROC curve; PPI, prepulse inhibition of the auditory startle reflex.

The described changes in frequency coding could, potentially, have different effects on behavioral measures of frequency discrimination. We next tested this using a behavioral measure of spontaneous frequency discrimination. We used the prepulse inhibition of the auditory startle reflex (PPI), a behavioral assay that is known to engage the inferior colliculus [34,35] and has been successfully used to determine frequency discrimination acuity in mice in the absence of training (Fig 6A). When assessed in the presence of a constant background tone, the percentage of PPI is proportional to the difference between the background and prepulse tones [36–38]. Predictable and random groups were exposed as before to a 16 kHz tone for 6–12 days in the Audiobox. PPI was then measured in a separate apparatus, using a background tone of 16 kHz and progressively different prepulse tones up to 1 octave (see Methods). The percentage of PPI elicited was significantly smaller in the predictable group than in the control and random groups at multiple prepulse frequencies tested (Fig 6B). Similarly, the average discrimination threshold (50% of inhibition of maximum response, see Methods) of the predictable group was higher than both the control and random groups but only reached significance against the latter (S5D Fig). The increased generalization in the predictable group was not specific to frequencies around 16 kHz. PPI measured with a background tone of 11.3 kHz in animals exposed to 16 kHz (Fig 6D) also showed a significant increase in frequency generalization (Fig 6E). Thus, only predictable sound exposure resulted in greater frequency generalization.

Next, we questioned whether changes in behavioral frequency discrimination were related to the collicular changes observed in frequency coding described above. We calculated ROC curves from the PPI data to be able to compare the behavioral and neuronal responses under the same method [31]. Surprisingly, the predictable and random groups showed larger AUROCCs when the background tone was 16 kHz, although the effect was not significant (Fig 6C). This is surprising because lower PPI is typically attributed to decreased discrimination acuity. The effect was specific to the frequencies around the exposed tone. When the background tone was 11.3 kHz, the increased generalization observed in the PPI for the predictable group was paralleled by diminished discrimination, as reflected in the lower AUROCCs, in this group with respect to the control group (Fig 6F).

In conclusion, the increased generalization observed in the PPI in the predictable group is consistent with the ROC analysis of the structural but not the local tuning for the same group (Fig 5F and 5G). This increase in generalization paradoxically did not reflect a decrease in discrimination, which was normal in both predictable and random groups for frequencies in the tuned region. That this effect was frequency specific, since discrimination was reduced for frequencies in the adjacent region, is consistent with the physiological classification accuracy measures (Fig 5D, 5E and 5I).

### Corticofugal input has a minor role on collicular plasticity induced by predictable sound exposure

**Fig 7:**
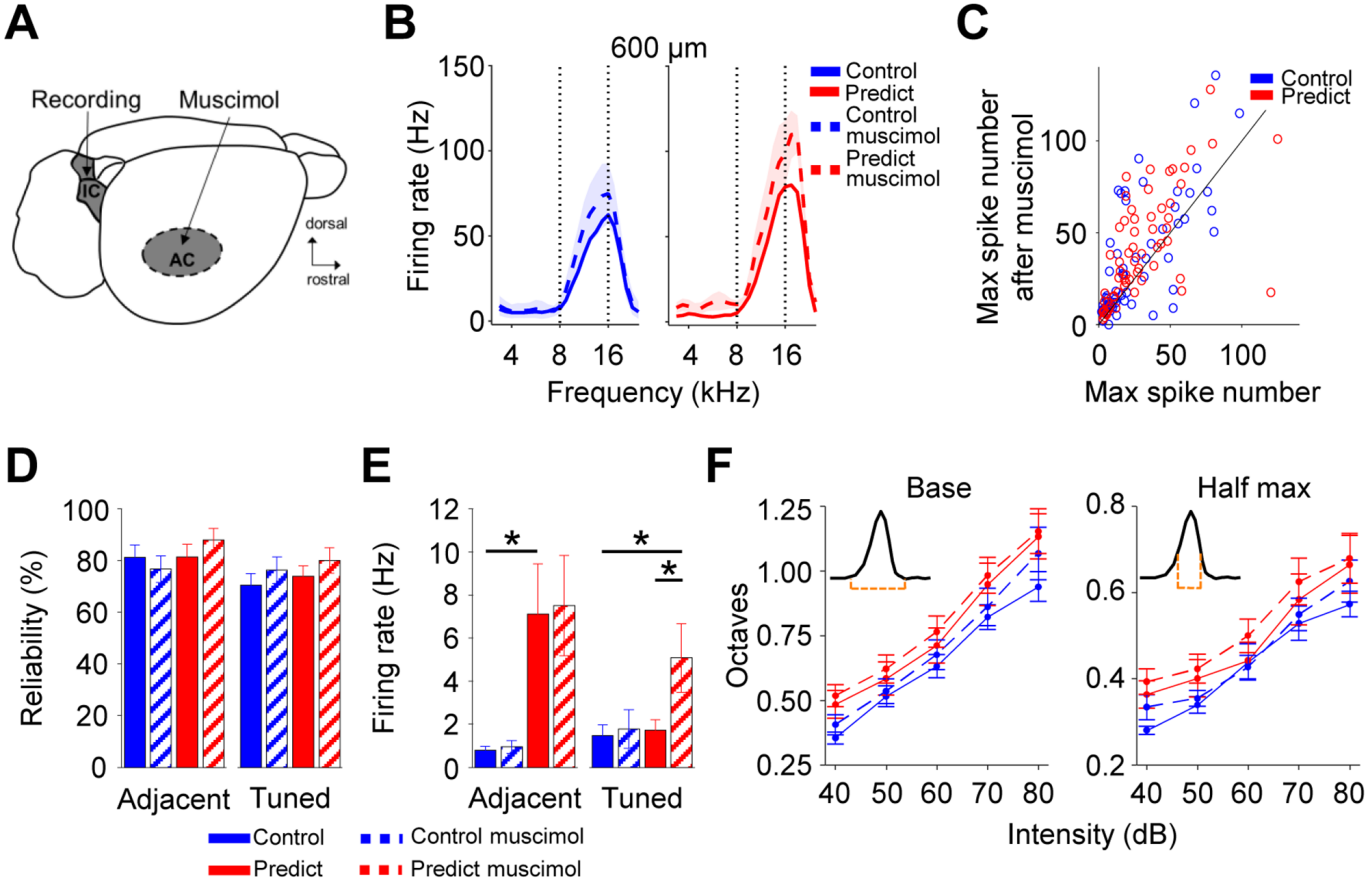
Cortical feedback does not influence sound exposure–induced collicular plasticity. (A) Schematic representation of simultaneous collicular recordings and cortical inactivation. (B) Average tuning curves at 600 μm for control (left) and predictable (right) groups before (continuous lines) and after cortical inactivation (dashed lines). (C) Pairwise comparison between activity before and after cortical inactivation (wilcoxon rank sum test, *p* = 0.4). Animals and recording sites: control *n* = 7 and 62, predictable *n* = 6 and 64. (D) Mean response reliability for the adjacent (left, ANOVA, group F_1,88_ = 1.22, *p* = 0.27) and tuned (right, ANOVA, group F_1,90_ = 1.62, *p* = 0.2) areas, before and after cortical inactivation. (E) Mean spontaneous activity for the adjacent (left, ANOVA, group F_1,92_ = 13.23, *p* < 0.001) and tuned (right, ANOVA, group F_1,94_ = 3.98, *p* < 0.05. Corrected pair comparisons: *p < 0.05) areas, before and after cortical inactivation. (F) Mean bandwidth as a function of sound intensity measured at the base (left, ANOVA, group F_1,229_ = 0.71, *p* = 0.4; muscimol F_1,229_ = 9.17, *p* < 0.01) or at the half-maximum (right, ANOVA, group F_1,229_ = 0.76, *p* = 0.38; muscimol F_1,229_ = 4.86, *p* < 0.05) of the tuning curve before and after cortical inactivation. Animals and recording sites: control *n* = 7 and 30; predictable *n* = 6 and 34. Error bars represent SEM. Numerical data for this figure found in S1 Data. AC, auditory cortex; IC, inferior colliculus.

Auditory conditioning studies have shown that collicular plasticity depends on direct cortical feedback through descending projections from layer V of the AC [39,40]. To test whether the maintenance of the changes in collicular response that had been triggered by predictable sound exposure were also dependent on cortical feedback, we performed simultaneous inactivation of the AC with muscimol and recordings in the inferior colliculus on a subset of control and predictable animals (see Methods, Fig 7A and S6A Fig). Cortical inactivation generated an increase in collicular evoked activity in both groups without affecting the differences in overall tuning between groups, including the BF shift (see tuning curves at 600 μm in Fig 7B; and S6B and S6C Fig). The increase in the activity of individual recording sites before and after cortical inactivation was comparable between groups (Fig 7C). Cortical inactivation affected neither reliability (Fig 7D) nor the difference in spontaneous activity in the adjacent region (Fig 7E, left). However, upon cortical inactivation, spontaneous activity of the predictable group increased in the tuned region (Fig 7E, right). This increase reveals a cortical control of collicular excitability that occurs specifically in the region tuned to the exposed sound. Cortical inactivation slightly increased the bandwidths for both groups without affecting the difference between them (Fig 7F). In summary, cortical inactivation resulted in an overall increase in the amplitude of the tuning curves that did not affect the difference in gain between the groups. The relatively lower spontaneous activity in the tuned region disappeared after cortical inactivation, revealing a frequency-specific form of cortical control on the inferior colliculus SNR. These data suggest that cortical feedback plays a minor role in the maintenance of sound exposure–triggered collicular plasticity.

### Predictable exposure does not lead to changes in the cochlear nucleus or AC

**Fig 8:**
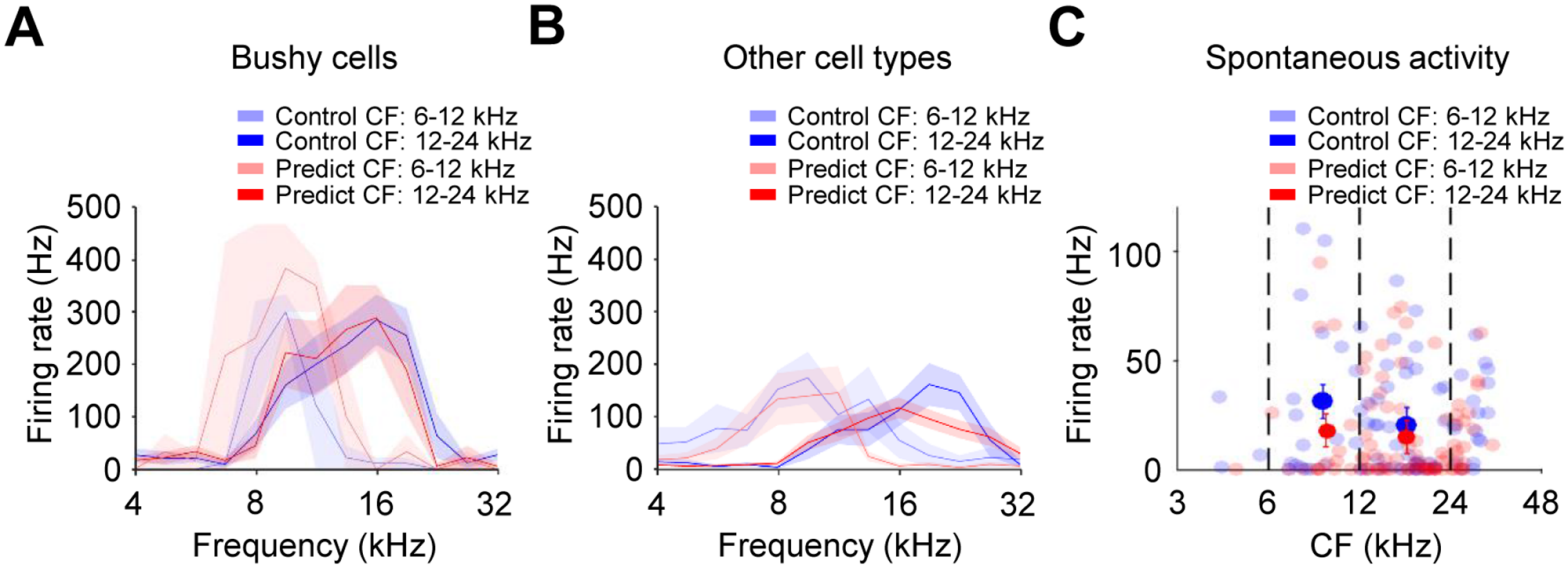
Predictable sound exposure does not affect evoked activity in the cochlear nucleus. (A) Average frequency response areas of cochlear nucleus neurons evoked by 70 dB tone bursts, classified as bushy cells. Units with a CF between 6 and 24 kHz were grouped by CF into 2 octave bins (CF group 6–12 kHz, control *n* = 3, predictable *n* = 2; and CF group 12–24 kHz, control *n* = 11; predictable *n* = 6; wilcoxon signed rank test, *p* > 0.05 for all comparisons). (B) Same as in A but for other cell types, mostly unipolar (CF group 6–12 kHz, control *n* = 9, predictable *n* = 11; and CF group 12–24 kHz, control *n* = 19, predictable *n* = 39; wilcoxon signed rank test, *p* > 0.05 for all comparisons). (C) Spontaneous firing rate distributions of cochlear nucleus units were comparable between control and predictable group (binning as in A-B, two-sample Kolmogorov-Smirnov test, *p* > 0.05 for all comparisons). Error bars represent SEM. Numerical data for this figure found in S1 Data. CF, characteristic frequency.

We next asked whether the changes in evoked activity and frequency representation were the result of an overall increase in excitability throughout the auditory pathway. Single-unit recordings in the cochlear nucleus—the main ascending input into the inferior colliculus—of animals in the control and predictable groups were similar in tuning, evoked, and spontaneous activity (Fig 8A–8C). Additionally, predictable sound exposure had no effect on either thresholds or bandwidths (S7A–S7D Fig), suggesting that exposure-triggered changes in the inferior colliculus were not the result of upstream plasticity. Similarly, evoked responses recorded in the primary auditory cortices of control and predictable mice were similar in overall tuning, temporal response pattern, and BF distribution (S7E–S7H Fig). Changes observed in the inferior colliculus were thus not inherited from the main upstream input, the cochlear nucleus. They also did not result in an obvious change in cortical tuning, although it is possible that more subtle effects would be observable in a behaving animal.

### Predictable sound exposure results in long-lasting changes in postsynaptic excitation/inhibition balance

Fast neuronal adaptation, previously described in the inferior colliculus [3,41], occurs within tens of seconds and would not necessarily be expected to be accompanied by changes in gene or protein expression. Sparse sound exposure, however, requires the integration of information across minutes and over several visits to the context associated with the sound. To investigate whether the observed changes were paralleled at the molecular level after predictable exposure, our key experimental condition, we measured gene expression in the predictable and control groups, using the home cage group as reference. We assessed the expression of neuronal genes reported to change their expression levels upon sound exposure, acoustic learning, or environmental enrichment [42–47]. In most cases, the expression was similar between the control and predictable groups and different from the home cage group (S1 Table), suggesting that the largest effect was triggered by the placement of animals in the Audiobox itself. Exceptions were the α-amino-3-hydroxy-5-methyl-4-isoxazolepropionic acid (AMPA) receptor subunits *gria1* and *gria2* and brain-derived neurotrophic factor *(BDNF)*, which were significantly reduced only in the control group with respect to the home cage group. The ratio between the expressions of the presynaptic markers glutamate vesicular transporter 2 (*vglut2*) and the GABA vesicular transporter (*vgat*) showed a significant increase for control and predictable groups.

To investigate whether the increase in the Vglut2/VGAT ratio at the level of gene expression were accompanied by molecular changes in protein expression at specific locations of the inferior colliculus, we measured immunoreactivity to VGAT and Vglut2 proteins at two depths (300 and 600 μm), corresponding roughly to the “adjacent” and “tuned” areas used before, in the central nucleus of the inferior colliculus of control and predictable animals (S8A Fig, see Methods). This ratio was used as an expression of excitation/inhibition balance, since this ratio has been shown to change upon environmental manipulations and to be a signature of synaptic plasticity [46]. We found that the number of Vglut2 puncta in the dorsal (“adjacent”) area was similar between groups, while VGAT was significantly reduced in the predictable group. This resulted in a significant increase in the Vglut2/VGAT ratio (S8B Fig, left). At 600 μm in depth (“tuned”), there was a decrease in Vglut2 in the predictable animals but only a trend in the same direction for VGAT, with no difference in the Vglut2/VGAT ratio between groups (S8B Fig, right). Thus, predictable and sparse sound exposure results in changes in gene and protein expression that are characteristic of long-term memory.

## Discussion

Statistical learning is essential for a correct interpretation of the sensory input. This form of learning is likely to be distributed throughout different brain regions, depending on the stimulus patterns to be learned, their modalities, and spatiotemporal combinations [48–50]. Some forms of statistical processing must happen at the level of subcortical structures as part of sensory gating. Neuronal adaptation—changes in firing rate as a result of continuous stimulation—is maybe the best-studied mechanism of experience-dependent plasticity believed to be underlying statistical learning of environmental regularities that occur within the recent stimulation history. It has been hypothesized to increase the dynamic range of neurons as well as gating of specific inputs [51] and is observed in cortical [2,7, 52–54] and subcortical structures [2–4]. Meta-adaptation has been observed across 5-second windows in a continuously alternating sensory stimulation paradigm in the inferior colliculus [4]. Yet the circuits underlying statistical learning of temporally sparse patterns have not been characterized. This timescale of statistical learning is reflected in the sensitivity of neurons in the auditory system for natural sounds [12–14, 55–58]. Neuronal adaptation is achieved through short-term plasticity [59–61]; therefore, it is unlikely to be the mechanism underlying the type of statistical learning that needs to be accumulated across bouts of exposure that are separated by minutes to hours, like the one we describe here.

Using a combination of electrophysiological, behavioral, and molecular approaches, we show that the inferior colliculus, an auditory subcortical structure, was sensitive to statistical learning of temporally sparse auditory patterns. We exposed mice to sounds that were fully predictable (predictable group). This exposure was self-initiated, limited to visits to the water corner (context specific), and lasted only for the duration of the individual visits (temporally sparse). Exposure to these patterns resulted in an increase in response gain that was frequency unspecific and was not due to mere sound exposure, since the random group (exposed to a sound in a fixed context but at random time intervals) showed a different pattern of collicular plasticity. Increase in response gain changed the pattern of population activity, resulting in increased between-frequency overlap in the structural tuning but a more consistent trial-to-trial within-frequency coding. These effects were paralleled at the behavioral level, at which increased response generalization was, paradoxically, not paralleled by a decrease in frequency discrimination as is discussed below. Cortical feedback played a minor role in the maintenance of collicular plasticity, and changes were not observed in the main input structure, the cochlear nucleus [62,63]. This suggests that plasticity was initiated in the inferior colliculus, as further supported by changes in gene expression indicative of long-term plasticity.

The combined analysis of local (region-specific tuning curves) and global (structural tuning) neuronal responses allowed us to uncover 2 coexisting mechanisms of frequency coding in the predictable group. On one hand, consistency in frequency coding was increased, as reflected in frequency-specific increase in classification accuracy. On the other hand, the potential for increased generalization was reflected in the increased overlap between structural tuning curves in the predictable group. Both increased discrimination and increased generalization were paralleled at the behavioral level. While, typically, a decrease in PPI has been interpreted as a decrease in frequency discrimination, here we found that different prepulse tones can generate discriminable startle responses and yet be less effective in generating PPI near the background tone. Thus, at the behavioral level, increased generalization in the startle’s inhibition was found to coexist with normal frequency discrimination near the exposed frequency. This highlights the relevance of responses across spatially distributed neuronal populations, in which even increased responses away from the tuned region (the tail of the structural tuning) might have an impact on behavioral output. Predictable sounds, when highly repetitive and consistent, are less salient. It is maybe because of this that behavioral responses to pure tones are largely more inhibited in the predictable group. In striking contrast, mice in the random group showed no evidence of diminished discrimination at either the neuronal population level or behaviorally, probably reflecting the saliency of randomness. Indeed, in this group, changes in response gain were—unlike in the predictable group—typically constrained to the tuned region.

Corticocollicular projections are believed to modulate collicular sensory filters [23,64–67]. The narrow corridors of the Audiobox prevented us from optogenetically modulating cortical activity during the exposure. Cortical inactivation during the recording, however, subtly increased the size of the evoked responses in both control and predictable groups and had no effect on either the suprathreshold tonotopic shift induced by sound exposure or the increase in bandwidth. However, it affected the levels of spontaneous activity. The frequency-specific low level in spontaneous activity in the tuned region disappeared upon inactivation, meaning that the cortical feedback can locally reduce spontaneous activity in one region of the inferior colliculus to increase the SNR. Nonetheless, overall, the cortical inactivation data suggest that the AC plays a small role in the maintenance of learning-induced plasticity and that this is limited to local modulations of spontaneous activity. Whether corticofugal feedback is required to initiate this plasticity in the early times of exposure will require further investigation.

Recently, Slee and David [68] reported increases in spontaneous activity in the inferior colliculus that resulted in suppression of responses to the target sound during an auditory detection task. Differences in excitability can be attributed to changes in interactions within the local circuit. In the predictable group, we observed changes in excitation/inhibition ratios at the presynaptic level that had no parallel at the postsynaptic level. Together, this might reflect the implementation of a switch that can be either turned on or off depending on, for example, the presence of a global signal in the form of a neuromodulator or brain state [69,70]. Indeed, a frequency-specific decrease in spontaneous activity in the predictable group resulted in an increase in SNR (evoked/spontaneous activity). SNRs have been studied in the context of speech saliency in noisy backgrounds [71–73] and have been hypothesized to contribute to compromised sensory gating in neuropsychiatric diseases, highlighting their importance for auditory processing [74]. Recordings were performed in anaesthetized animals, and although anesthesia does not prevent the expression of preattentive mechanisms, the exact implementation of the proposed switch might be different in the behaving animal [75,76].

In both exposed groups, we observed a surprising shift in suprathreshold tonotopy with respect to the control group. This was reflected in a homogeneous shift in BFs across all depths measured. This shift was significantly larger in the predictable group than in the random group. While reinforcement-driven plasticity is characterized by locally measured shifts toward a conditioned frequency in both inferior colliculus and AC [77,78], spatially broad frequency shifts cannot always be measured. In the one case in which this was done [64], the shift was also found to extend beyond the directly activated frequency band. Whether the inferior colliculus uses the BF shift as a coding mechanism or this is rather a byproduct of other plastic changes will require further investigation. In fact, BF might not be a very reliable coding variable [79,80]. Measurements such as structural tuning, in which simultaneous responses across a widespread neuronal population are measured, might better represent the information that the brain is using at any given point in time.

Differences in sensory filtering at the level of the inferior colliculus are likely to influence how information is conveyed downstream to thalamus and cortex. Depending on whether the change impinges primarily on the excitatory or inhibitory ascending input into the thalamus, the overall effect might be either to enhance or suppress selective responses. The collicular inhibitory input into the thalamus acts monosynaptically on thalamocortical projecting neurons [81], potentially regulating the magnitude and timing of cortical activity and thus playing a crucial role in sensory gating. We did not find obvious changes in excitability or frequency representation at the cortical level after predictable sound exposure. In the auditory system, which processes a constant input of stimuli arising from all directions, preselection of to-be-attended stimuli might happen at the level of subcortical structures. In other sensory systems, filtering of stimuli might involve different circuit mechanisms [82,83].

Taken together, our results demonstrate that the inferior colliculus, a subcortical structure, plays a significant role in the detection of statistical regularities that arise from temporally sparse interactions with a naturalistic environment. The effect this learning had on subsequent behavior suggests that the observed changes in coding modulate the filtering of the exposed sounds to control behavioral outcomes. Our study places the inferior colliculus as a key player in the processing of context–sound associations, which are of great relevance in sound gating. This role might be the basis for the link between the inferior colliculus and autism, in which patients exhibit alterations in sensory gating [84–86]. The finding that neuronal responses are sensitive to the context in which sounds appear suggests that the inferior colliculus might integrate stimuli across a parameter space that goes beyond the auditory domain. Thus, the inferior colliculus could be acting as an early multimodal warning system.

## Methods

### Ethics statement

All animal experiments were approved by the local Animal Care and Use Committee (LAVES, Niedersächsisches Landesamt für Verbraucherschutz und Lebensmittelsicherheit, Oldenburg, Germany) in accordance with the German Animal Protection Law. Project license number 33.14-42502-04-10/0288 and 33.19-42502-04-11/0658.

### Experimental model and subject details

Female mice C57BL/6JRj (Janvier labs, France) between 5 and 8 weeks old were used for all experiments.

### Audiobox

A sterile transponder (IS0 compliant 11784 transponder, 12 mm long, TSE, Germany) was implanted subcutaneously in the back of the anaesthetized mice. The small wound caused by the injection was closed with a drop of a topical skin adhesive (Histoacryl, Braun, United States of America). After 1 to 2 days of recovery, animals were placed in the Audiobox (New Behaviour/TSE, Germany).

The Audiobox is an automatic testing chamber consisting of 2 compartments connected by a corridor (Fig 1A), where mice lived in groups of up to 10 animals. The first compartment—the “food area”—consists of a normal mouse cage, where animals have ad libitum access to food. Water was available in the second compartment—the “water corner”—located inside a sound-attenuated chamber. An antenna located in the entrance of the corner identified the individual mouse transponder. The individual visits to the corner were detected by coincident activity of a heat sensor and the reading of the transponder. Visits occurred mainly during the dark cycle [15]. A water port is present at either side of the corner and can be closed by a sliding door. To open the door and gain access to the water, animals needed to nose-poke. Nose-pokes were detected by a sensor located by the door. The end of the visit was signaled by deactivation of the heat sensor and the absence of transponder reading. Individual-mouse data (start and end of visit, time and number of nose-pokes) were recorded for each single visit. Visits to the corner could be accompanied by a sound, depending on the identity of the mouse. A loudspeaker (22TAF/G, Seas Prestige) was located above the corner to present sound stimuli. The sounds presented were generated in MATLAB (The MathWorks, USA) at a sampling rate of 48 kHz and consisted of 30 ms pure tones with 5 ms slope, repeated at 3 Hz for the duration of the visit and at variable intensity of 70 dB ± 5 dB (measured at the center of the corner in the predictable group or the center of the home cage in the random group). The sound intensity was calibrated with a Bruël & Kjaer (4939 ¼” free field) microphone. The microphone was placed at different positions within the corner, as well as outside the corner, while pure tones (1–40 kHz) were played at 60–70 dB. Microphone signals were sampled at 96 kHz and analyzed in MATLAB. Tones between 3 kHz and 19 kHz did not show harmonic distortions within 40 dB from the main signal. The sounds presented inside the corner were attenuated by over 20 dB outside the attenuated box. Since little attenuation occurred in the corridor located inside the attenuated box immediately connected to the corner, mice in this location could hear the sound played in the corner.

### Sound exposure

All the experimental groups were first habituated to the Audiobox for 3 days without sound presentation. After the habituation phase, the exposed group heard a fixed-tone pip of a specific frequency for the duration of every visit, regardless of nose-poke activity and water intake. The random group was exposed to a fixed-tone pip in the mouse cage at random intervals. The sound was delivered by a loudspeaker located above the cage and calibrated such that sound intensity in the center of the cage was comparable to that inside the corner. The presentation of the sound was triggered by corner visits of a mouse living in another Audiobox, in a yoke control design. This ensured that the pattern (mainly at night) and duration of sound presentation in the cage was comparable to that experienced by each mouse in the predictable group when making corner visits. The control group consisted of age-matched animals that lived during the same amount of time in a different Audiobox without sound presentation. The number of mice reported in Fig 1C–1E corresponds to exposed animals to 16 kHz used for recordings in the inferior colliculus and AC. The sounds used during the exposure phase were fixed for each mouse and replication: 8, 13, or 16 kHz, depending on the experiment. One group of animals (8 and 13 kHz group) was exposed in 71% of the visits to 8 kHz and the remaining 29% of the visits to 13 kHz, similar to the preconditioned phase of the LI protocol.

### LI

The experiment consisted of 4 phases: habituation, safe, exposure, and conditioning [15]. Animals were divided in 3 different groups that differed only in the exposure phase before conditioning. During the habituation phase (3 days), no sound was presented, and the sliding doors remained open. In the safe phase (7 days), a safe tone of 8 kHz was paired with every visit to the corner, and the sliding doors opened only after nose-poke. In the exposure phase (5 days), groups were exposed to different frequencies as follows: (i) for the control group, 71% of the visits were paired with an 8 kHz tone, and 29% were paired with a 4 kHz tone; (ii) for the predictable group, 71% of the visits were paired with an 8 kHz tone, and 29% were paired with a 16 kHz tone; (iii) for the random group, 100% of the visits were paired with 8 kHz, and a 16 kHz tone—played in the home cage—was paired to 29% of the visits of a mouse living in another Audiobox to its corresponding corner. Up to this point, all nose-pokes resulted in access to water independently of the sound played. In the conditioning phase, 71% of visits were paired with an 8 kHz tone, and 29% were paired with a 16 kHz, which was conditioned such that a nose-poke resulted in an air puff and no access to water. During this phase, mice had to learn to avoid nose-poking when they heard 16 kHz (conditioned visit). To assess discrimination performance, the discriminability index (d’) was calculated. d’ used in signal detection theory is defined as

$$d^{'}=Z(HR)-Z(FAR),$$

in which *Z*(*p*), *p* ∈ [0 1] is the inverse of the cumulative of the gaussian distribution; HR is the hit rate, in which a hit is the correct avoidance of a nose-poke during a conditioned visit; and FAR is the false alarm rate, in which a false alarm is the avoidance of a nose-poke during a safe visit. Since d’ cannot be calculated when either the hits or the false alarms reach levels of 100% or 0%, in the few cases when this happened, 99% and 1%, respectively, were used for these calculations.

### Electrophysiology

Mice were anesthetized with avertin before acute electrophysiological recordings in the inferior colliculus (induction with 1.6 mL/100 grs and 0.16 mL/100 grs ip to maintain the level of anesthesia as needed). Anesthetized mice were fixed with blunt ear bars on a stereotaxic apparatus (Kopf, Germany). The temperature of the animal was monitored by a rectal probe and maintained constant at 36 °C (ATC 1000, WPI, Germany). The scalp was removed to expose the skull, and bregma and lambda were aligned vertically (± 50 μm). A metal head-holder was glued to the skull 1.3 mm rostral to lambda to hold the mouse, and the ear bars were removed. To access the left inferior colliculus, a craniotomy of 2.8 × 3 mm was made, with the center 1 mm lateral to the midline and 0.75 mm caudal to lambda. The inferior colliculus was identified by its position posterior to the transverse sinus and anterior to the sigmoid sinus.

The tip of the left inferior colliculus became visible after the craniotomy, and measurements from the rostrocaudal and mediolateral borders were made to place the recording electrode exactly in the middle of the inferior colliculus, targeting the central nucleus. The probe was inserted such that the most dorsal electrode was aligned with the dura (Fig 2B), thus minimizing the error in depth alignment. An error in depth assessment might arise from the topmost recording site (with a diameter of 13 μm) not being exactly aligned with dura. Since the electrode sites are visible under microscope, the depth error is unlikely to have been more than ± 25 μm (half the distance between electrode sites). Other measures were in place to ensure reliability of the positioning: (1) before inserting the probe, bregma and lambda were aligned to the same horizontal plane; (2) the probe was lowered at a fixed rostrocaudal and mediolateral position with respect to bregma; (3) the probe angle was 90° with respect to the bregma–lambda plane; (4) dura was intact; and (5) penetration was very slow. Extracellular multiunit recordings were made using mainly multielectrode silicon arrays (Neuronexus Technologies, USA) of 16 electrode sites in either a single shank (most data; 177 μm^2^ area/site and 50 μm spacing between sites) or 4 shanks (rostrocaudal analysis; 150 μm intershank spacing). Glass-coated single electrodes were used to collect data on exposure to frequencies other than 16 kHz. These were either glass-coated tungsten electrodes with a typical impedance of 900 mOhm and an external diameter of 140 μm (AlphaOmega, Germany) or glass-coated platinum/tungsten electrodes with a typical impedance of 1 mOhm (Thomas Recordings, Germany). The electrodes were inserted in the central part orthogonally to the dorsal surface of the inferior colliculus and lowered with a micromanipulator (Kopf, Germany). In the case of single electrodes, recordings were made every 50–100 μm. When multielectrode silicon arrays were used, they were lowered (at a rate of 100 μm/5 minutes) until the upper electrode was in contact with the inferior colliculus surface, visualized with a microscope (750 μm depth). The electrodes were labeled with DiI (1,1'-dioactedecyl-3,3,3,3'-tethramethyl indocarbocyanide, Invitrogen, Germany) to allow the reconstruction of the electrode track in postmortem sections using standard histological techniques (Fig 2B).

### Data acquisition

The electrophysiological signal was amplified (HS-36 or HS-18, Neuralynx, USA) and sent to acquisition board (Digital Lynx 4SX, Neuralynx, USA). The raw signal was acquired at 32 kHz sampling rate, band-pass filtered (0.1–9,000 Hz), and stored for offline analysis. Recording and visualization were made by Cheetah Data Acquisition System (Neuralynx, USA).

### Acoustic stimulation during electrophysiological recordings

The sound was synthesized using MATLAB, produced by an USB interphase (Octa capture, Roland, USA), amplified (Portable Ultrasonic Power Amplifier, Avisoft, Germany), and played in a free-field ultrasonic speaker (Ultrasonic Dynamic Speaker Vifa, Avisoft, Germany) located 15 cm horizontal to the right ear. The sound intensity was calibrated at the position of the animal’s right ear with a Bruël & Kjaer (4939 ¼” free field) microphone. Microphone signals were sampled at 96 kHz and analyzed in MATLAB. Tones between 2 kHz and 30 kHz did not show harmonic distortion within 40 dB from the main signal. Sound stimuli consisted of 30 ms pure-tone pips with 5 ms rise/fall slope played at a rate of 2 Hz. We used 24 frequencies (3.3–24.6 kHz, 0.125 octave spacing) at different intensities (0–80 dB with steps of 5 or 10 dB) played in a pseudorandom order. Each frequency-level combination was played 5 times. For the analysis of SNRs, data were bundled in “adjacent” and “tuned” regions. Each of these regions comprised 4 steps in the frequency sweep (14.6, 16, 17.6, and 19 kHz for the tuned; 10.3, 11.3, 12.3, and 13.4 kHz for the adjacent region) and ranges of frequencies with a ΔF of 30%. For the two-tone inhibition protocol, a fixed tone (16 kHz, 50 dB) was played simultaneously with a variable tone of a specific frequency-intensity combination (3.3–24.6 kHz, 0.125 octave spacing; 0–80 dB with steps of 5 or 10 dB).

### Analysis of electrophysiological recordings

The stored signals were high-pass filtered (450 Hz). To improve the SNR in the recordings with the silicon probes, the common average reference was calculated from all the functional channels and subtracted from each channel [87]. Multiunit spikes were then detected by finding local minima that crossed a threshold that was 6 times the median absolute deviation of each channel (S2A Fig). Recorded sites were classified as sound driven when they fulfilled 2 criteria: (1) Significant evoked responses: a PSTH was built, with 1 ms bin size, combining all the frequencies and the intensities above 30 dB. The overall spike counts over 80 ms windows before and after tone onset were compared (*p* < 0.05, unpaired *t* test). (2) Responses were excitatory: they crossed an empirically set threshold (evoked spikes–baseline spikes) of 45 spikes. Responses that were inhibitory (less evoked spikes than baseline, <10% of cases) were not used. Using these criteria, 85% of the recorded sites where classified as sound driven.

In auditory-driven recording sites and for each testing protocol, the spikes across all the trials for each frequency-intensity combination were summed at 1 ms bins. Evoked firing rates were calculated in an 80 ms window, starting with stimulus onset expressed as spikes per second. This yielded a specific spike rate per each frequency-intensity combination that was used to build iso-intensity tuning curves. The peak in collicular activity for each group was computed by averaging the peak of the tuning curve at 70 dB for each recording site along the tonotopic axis.

The BF (frequency that elicited the best response in a given recording depth) was selected as that with the highest spike count when responses were summed over all intensities. In the rare cases in which more than one frequency elicited the highest response, the mean was used as BF. The difference in BF along the tonotopic axis was computed as the mean across depths of each individual BF minus the average control BF at each depth.

Reliability was calculated for recording sites with a BF within a specific range. For each selected site, reliability was calculated as the percentage of trials in which the BF in the selected range evoked at least 1 spike at 70 dB. The spontaneous activity was calculated as the firing rate within a window of 80 ms previous stimulus onset. The SNR was the ratio between the activity evoked by a specific frequency at 70 dB (calculated as described above) and the spontaneous activity.

The intensity threshold—the lowest sound intensity that elicited a reliable response—was calculated from the FRA as the lowest sound intensity that elicited a spike count 1.5 times higher than the spontaneous activity [88].

The bandwidth at the base, for each sound intensity above threshold, was calculated from the smoothed FRA (4-point averaging [88]) as the width in octaves of the frequencies that evoked at least 20% of the maximum response. The bandwidth at half-maximum, for each sound intensity above threshold, was calculated from the smoothed FRA as the width in octaves of the frequencies that evoked 50% of the maximum response at each intensity level. Only recording sites with a BF of 9 to 16 kHz were included in the analysis to avoid the inclusion of incomplete tuning curves due to the frequency range we used as stimuli.

The intensity-specific BF corresponded to the frequency that elicited the strongest response at each sound intensity. Latencies corresponded to the time after sound offset of the first evoked spike.

### ROC analysis

ROC analysis was used to assess the discriminability across frequencies in the tuning curves, across structural tuning curves, and across prepulse frequencies in the behavioral PPI.

For the tuning curves (local tuning), we generated response distributions (perfcurve function, MATLAB) based on the number of spikes elicited by a given tone across trials (Fig 5A left). The probability that a given frequency f2 will be bigger than a growing criterion of number of spikes will go from 1 to 0 as the criterion traverses the range of spike numbers elicited by f2 (Fig 5A right). For the blue f2 in the figure, the criteria that elicit probabilities above 0 will overlap with those of f1 (yellow), while for the brown f2, there will be no overlap. The ROC curve will therefore be largest for the comparison between the brown f2 and f1 and shallower for the comparison between the blue f2 and f1 (Fig 5B).

The ROC analysis of the structural tuning was based on the variability in the size of the response across depths (250 to 750 μm), rather than trials, and was calculated for structural tuning curves elicited by individual tone presentations (trials, Fig 5C). The number of spikes was used to generate depth distributions in the same way that the number of trials was used to generate spike distributions for the local tuning. In this case, f1 was either the average structural tuning of 16 kHz or 11.3 kHz, while f2 was the trial-by-trial structural tuning of frequencies below f1. The trial-by-trial ROC values for each frequency were averaged before they were plotted.

The ROC analysis for the behavioral data was based on the variability in the startle response across prepulse presentations of a given frequency (see PPI methods below). Distributions were constructed, like for the local tuning, from the individual trial values. For each PPI test, f1 was whatever frequency was the background frequency (16 or 11.3 kHz), and f2 varied across the range of prepulse frequencies.

### Classification accuracy model

Structural tuning–based classification [32,33] was performed as follows. The input to the model is a spike-counts dataset of size S × T × *N* in which S is the total number of stimuli (S = 24 frequencies), T is the number of repetitions for each stimulus (T = 5), and *N* is the number of recorded depths (*N* = 14). The vector V^s,t^ = (V^s,t^_1_,…,V^s,t^_N_) represents a single-trial response of the neural population to stimulus s, in which s goes from 1 to S, and t goes from 1 to T. The model is then “trained” to create individual response templates for each stimulus s calculated by averaging the vector V^s,t^ over the T − 1 trials in the training set. The single trial left out of the training set is used to generate a prediction and classified as being generated by a given stimulus if the euclidean distance between the single trial and the template corresponding to that stimulus is minimal compared to all the other distances. We classified all S × T single trials using this scheme and summarized the results in a confusion matrix C of size S × S, in which the i,j-th element C_i,j_ is the fraction of trials with stimulus i being classified as stimulus j. The individual confusion matrices, representing the probability of correctly predicting the actual frequency, were averaged across groups and used to estimate classification accuracy.

### PPI

Animals were placed in a custom-made acrylic chamber of 12 cm long and 4 cm in diameter. Movement was detected by a piezoelectric sensor located below the chamber. The protocol was as previously reported by others [36,37].

The experiment was divided in 5 phases following one after the other uninterruptedly. (1) Chamber habituation: at the start of each session, animals were placed in the test chamber and allowed to habituate for 10 minutes; (2) Sound habituation: a constant background tone (f1: 16 kHz, 70 dB SPL) was played for 5 minutes; (3) Startle-only trials: 10 startle-only trials were presented on the background of 16 kHz to allow for short-term habituation to the startle sound; (4) Test phase: 10 pre-pulse trials and 10 startle only trials were presented to assess frequency discrimination; (5) Startle-only trials: 5 startle-only trials were presented to check for habituation over the duration experiment. Trials consisted of a frequency change from the background tone (f1) to the prepulse tone (f2, 80 ms long, 1 ms ramp) at constant 70 dB SPL (Fig 1F). This was immediately followed by 20 ms broadband noise (BBN) at approximately 100 dB, which was in turn followed by the background tone at 70 dB until the following trial in a seamless manner. For the “startle-only trials,” f1 and f2 were 16 kHz, and for prepulse trials, f2 was 15.92, 15.84, 15.68, 15.472, 15.2, 14.72, 14, or 8 kHz, corresponding to Δf of 0.5%, 1%, 2%, 3.3%, 5%, 8%, 12.5%, and 50%, respectively, relative to f1. For animals in which f1 was 11.3 kHz, f2 was 11.31, 11.25, 11.19, 11.08, 10.93, 10.74, 10.4, 9.89, or 5.65 kHz. Trials had pseudorandom lengths between 8 and 25 seconds.

The mouse acoustic startle reflex was measured as the maximal vertical force exerted on the piezo within a 200 ms window starting with the onset of the startle noise, minus the mean of the force for 50 ms before the startle noise. For each animal, the startle-only trials of the test phase and the prepulse trials of each frequency were averaged. The percent of PPI for each prepulse frequency PPI (%) was calculated as follows:

$$PPI(\%)=100\;\times \;\frac{ASRnopps\;-\;ASRpps}{ASRnopps},$$

in which ASRnopps is the mean response of the startle-only trials, and ASRpps is the mean response of the prepulse trials for that particular frequency. Discrimination thresholds for each animal, defined as the Δf that caused 50% of inhibition of the maximum response, were calculated from parametric fit to a generalized logistic function (fit function MATLAB) [37]

$$PPI=-\frac{a}{2}\;+\;\frac{a}{1\;+\text{exp}\;\left(b\;+\;c\Delta f\right)}.$$

Animals with a fit coefficient of the curve (R^2^) below 0.7 were excluded from statistical analysis (3 control animals, 2 exposed animals, and 1 random animal). Additionally, the pooled data for each group were also fitted to a generalized logistic function.

### Simultaneous cortical inactivation and recordings in the inferior colliculus

In a subset of the animals and after the surgery in the inferior colliculus, a 4x3 mm craniotomy medial to squamosal suture and rostral of the lambdoid suture was made to expose the left AC. The AC was located dorsal and posterior of the transverse sinus [89]. A small amount of Vaseline was applied to the boundaries of the craniotomy to form a well. A single electrode or a 16-channel multielectrode array was inserted. Evoked responses to the tone pips were constantly monitored. A small amount of volume of phosphate-buffered saline solution (Sigma, USA) was applied (3–5 μL) every 10–15 minutes during baseline recordings in the inferior colliculus. Then, 3–5 μL of muscimol were applied over the AC (1 mg/mL, dissolved in phosphate-buffered saline solution, Sigma, USA). AC evoked activity was monitored using frequency sweeps at 70 dB SPL or BBN of different intensities every 5 minutes. AC was usually inactivated 15–20 minutes after muscimol application. Once cortical inactivation was confirmed, recordings in the inferior colliculus were repeated.

### Single-unit recording from cochlear nucleus

Six to 12 days after the beginning of sound exposure (8 kHz), mice were removed from the Audiobox one at a time for acute electrophysiology. Mice were anesthetized with urethane (1.32 mg/kg, ip) and xylazine (5 mg/kg, ip). Animal temperature was maintained at 36.5 °C using a custom-designed heating pad in a soundproof chamber with ambient temperature of 30 °C. A tracheotomy was performed, and the cartilaginous ear canals were removed before the mouse was positioned in a custom-designed head-holder and stereotaxic apparatus. Then, a craniotomy was performed on part of the occipital bone, and part of the cerebellum was aspirated to visualize the superior semicircular canal as a reference point. A glass microelectrode filled with 2 M NaCl and 1% methylene blue was advanced in 4 μm steps (Inchworm micromanipulator, EXFO Burleigh, Germany), aiming for the anterior part of the anteroventral cochlear nucleus. Extracellular signals were amplified and band-pass filtered (300–3,000 Hz) using an ELC-03X amplifier (NPI Electronic, Tamm, Germany). Digitized signals (TDT system 3) were saved for offline analysis using custom-written MATLAB software. Once a sound-responsive neuron was isolated, the spontaneous rate, CF, and best threshold were determined as described by Jing and colleagues [90]. Unit classification was based on the response pattern to 200 repetitions of 50 ms tone burst at CF (2.5 ms cos^2^ rise/fall, 10 Hz repetition rate), as described by Taberner and Liberman [91]. The analysis for “other cell types” includes mostly chopper units, some onset units, and a few pauser/build-up units. Likewise, responses to 8 kHz tone bursts were recorded, and the receptive area of each unit was mapped using 30 ms tone bursts at 70 dB (10 repetitions per sweep, 3 Hz repetition rate) for a total of 13 frequencies ranging from 4 kHz to 30 kHz.

### AC recordings

A 4 × 3 mm craniotomy medial to squamosal suture and rostral of the lambdoid suture was made to expose the left AC. The AC was located dorsal and posterior of the transverse sinus [89]. Single-electrode penetrations (400–450 μm) were made along the exposed cortical surface spaced between 200–250 μm. Auditory core fields (A1 and AAF) were identified according to their response latencies and tonotopic distribution [89]. Data acquisition and acoustic stimulation were similar as with inferior colliculus recordings.

### Gene expression analysis

A separate set of mice was used for gene expression analysis. After 3 days of habituation and 7 days of sound exposure in the Audiobox, mice were anesthetized with avertin and killed by cervical dislocation; immediately, the brain was extracted; and both inferior colliculi were dissected and immediately frozen at −80 °C and stored for later analysis. RNA was isolated from inferior colliculi using the RNAeasy Kit (Qiagen), following manufacturer’s instructions. cDNA was synthesized from 1 μg of RNA using the Superscript III Kit (Invitrogen) and random nonamer primers. For quantitative real-time PCR, SyBr Green Master Mix kit (Applied Biosystems, Germany) was used, and amplification reactions were run on a Roche LC480 Detection System (384-well plates) or 7500 Fast Real-Time PCR System (96-well plates). Reactions were run in 4 replicates. The efficiency (*E*) of each pair of primers was estimated based on the slope (*m*) of a standard curve of the Ct values from 5 serial logarithmic dilutions of a template cDNA, using the following formula:

$$E=10^{\left(\frac{-1}{m}\right)}.$$

The goodness of fit (R^2^) of all the standard curves was >0.98.

We used the gene of the ribosomal protein L13a (*rpl13a*) as a reference gene, since it has been reported as the best candidate gene for brain gene expression analysis [92]. The relative expression of Rpl13a showed no change between the three groups tested (F_2,17_ = 0.8, *p* = 0.47, *n* = 7, 8, and 5 for exposed, control, and home cage groups, respectively).

Gene expression relative to the housekeeping gene (Rpl13a) was calculated with the method used by [93], in which corrections for different efficiencies between target gene and housekeeping gene are made:

$$RE=\frac{{Ekhg}^{CThkg}}{{Etg}^{CTtg}},$$

in which *RE* is the relative expression, *Ekhg* is the efficiency of the housekeeping gene, *CThkg* is the *Ct* value of the housekeeping gene, *Etg* is the efficiency of the target gene, and *CTtg* is the *Ct* value of the target gene.

### Statistical analysis

After testing for normality distribution using the Jarque-Bera test, group comparisons were made using multiple way ANOVAs, accordingly. For experiments with multiple measures per animal, we used mixed-design ANOVA, with mouse identity as a nested random effect. To test the effect of days on frequency representation and collicular activity, we used a linear mixed effects model (*fitlme*, MATLAB, with mouse identity as a random effect). For data in which normality test failed, a Kruskal-Wallis test or wilcoxon signed rank test for paired data was used. Where possible, post hoc Bonferroni corrections for multiple comparisons were used. Means are expressed ± SEM. Statistical significance was considered if *p* < 0.05.

## Supporting information

S1 Data Excel spreadsheet containing, in separate sheets, the underlying numerical data for figure panels Figs 1D–1E, 2C–2E, 3B–3C, 3B–3D, 3F–3G, 4C, 5D–5G, 5I, 6B–6C, 6E–6F, 7B–7F, and 8A–8C. (XLSX)

S2 Data Excel spreadsheet containing, in separate sheets, the underlying numerical data for figure panels S1A–B, S1EF, S2E, S3A–F, S4A–C, S5A–D, S6B–C, S7A–G, and S8B. (XLSX)

S1 Fig Predictable and random sound exposure have different effect on subsequent conditioning in the Audiobox. (A) Cumulative distribution of the visit duration to the water corner area. (B) Mean daily percentage of visits without NPs was similar between groups (ANOVA, F_2,60_ = 1.47, *p* = 0.23). (C) Scheme of the latent inhibition protocol. All phases were identical across groups except for the exposure phase. Colored boxes indicate the frequency of sound exposure (30% of visits). To avoid sound novelty effects, 8 kHz was used in remaining visits (safe visits). Conditioning took place only during the conditioning phase and only in visits in which 16 kHz was played (black bars). (D) Schematic representation of the conditioning phase in the latent inhibition paradigm. Safe visits were accompanied by an 8 kHz tone (left, gray color). Conditioned visits were accompanied by a 16 kHz sound, and NP on either side resulted in an air puff (right, orange color). (E) Mean discriminability index (d’) during the first day of conditioning was lower for the predictable group (ANOVA, F_2,49_ = 13.69, *p* < 0.01; ***p < 0.0001; **p < 0.001). Control *n* = 15; predictable *n* = 18; random *n* = 19. (F) Mean visits without NPs per day and group during the first 5 days of conditioning for the conditioning visits only. Error bars represent SEM. Numerical data for this figure found in S2 Data. NP, nose-poke.
(TIF)

S2 Fig Extracellular recordings in the inferior colliculus. (A) Representative examples of spike waveforms recorded from a given electrode at a given depth (300–600 μm) for the control (upper row), predictable (middle row), and random (lower row) groups. (B-D) Representative examples of raster plots recorded at 70 dB SPL at different depths from one control (B), one predictable (C), and one random animal (D). Each dot represents a spike and each line, one of 5 repetitions of a 30 ms tone. Vertical red lines indicate the onset and offset of the tone. (E) Individual tuning curves for animals in the control (blue), predictable (red), and random (green) groups for depths with BF of 16 kHz in the mice in the predictable and random groups. Numerical data for this figure found in S2 Data.
(TIF)

S3 Fig Predictable and random sound exposure increase evoked activity in different region of the inferior colliculus. (A) Mean firing rate per collicular zone (100–150 μm: ANOVA, group F_2,1080_ = 22.64, *p* < 0.0001; 200–300 μm: ANOVA, group F_2,1944_ = 15.21, *p* < 0.001; 350–450 μm: ANOVA, group F_2,1680_ = 9.54, *p* < 0.001; 500–600 μm: ANOVA, group F_2,1848_ = 21.46, *p* < 0.0001; 650–750 μm: ANOVA, group F_2,1512_ = 21.31, *p* < 0.0001. Corrected pair comparisons ***p < 0.0001, **p < 0.01). For A-B, D-E: animals and recording sites: home cage *n* = 6 and 72; control *n* = 10 and 98; predictable *n* = 14 and 162; random *n* = 7 and 91. (B) Mean maximum firing rate (ANOVA, group F_3,367_ = 4.2, *p* < 0.01; corrected pair comparisons: *p < 0.05). (C) Group mean tuning curves of responses at 200 μm of animals in control and 8 kHz–exposed predictable group (ANOVA, group × frequency interaction F_23,276_ = 4.22, *p* < 0.05). Control *n* = 10; 8 kHz *n* = 4. (D) Distribution of number of days in the Audiobox per group. Animals: control *n* = 10; predictable *n* = 14; random *n* = 7. (E) Peak response per mouse across days in the Audiobox per group as in (D). Overall ANOVA (group × days) revealed no effect of group F_2,22_ = 1.63, *p* = 0.22; or days F_6,22_ = 1.44, *p* = 0.25. There was no effect of days within each group: control, F_3,6_ = 2.62, *p* = 0.15; predictable, F_6,7_ = 3.06, *p* = 0.08; random, F_4,2_ < 1. (F) Tuning curves aligned by BF ± 0.05% for BFs with at least 4 mice/group. An overall ANOVA (group × tuning BF × frequency played) revealed an effect of group F_2,4752_ = 12.55, *p* < 0.001; BF F_9,4752_ = 10.01, *p* < 0.001; and frequency F_23,4752_ = 40.96, *p* < 0.001; and an interaction between group and BF F_18,4752_ = 12.81, *p* < 0.001; and BF and frequency F_207,4752_ = 5.21, *p* < 0.001. Within each BF range, all group comparisons revealed an effect of group: 9,510 Hz, ANOVA, group F_2,384_ = 15.46, *p* < 0.001; 10,370 Hz, ANOVA, group F_2,456_ = 4.91, *p* < 0.001; 11,310 Hz, ANOVA, group F_2,504_ = 4.98, *p* < 0.01; 12,340 Hz, ANOVA, group F_2,336_ = 1.51, *p* = 0.22; 13,450 Hz, ANOVA, group F_2,528_ = 7.54, *p* < 0.001; 14,670 Hz, ANOVA, group F_2,456_ = 8.15, *p* < 0.001; 16,000 Hz, ANOVA, group F_2,528_ = 7, *p* < 0.001; 17,450 Hz, ANOVA, group F_2,528_ = 4.93, *p* < 0.01; 19,030 Hz, ANOVA, group F_2,528_ = 3.34, *p* < 0.05; 20,750 Hz, ANOVA, group F_2,504_ = 99.84, *p* < 0.0001. Error bars represent SEM. Numerical data for this figure found in S2 Data. BF, best frequency.
(TIF)

S4 Fig Predictable sound exposure induces a shift in frequency representation along the inferior colliculus. (A) Across depth mean difference in BF with respect to mean BF of control group for groups exposed to frequencies other than 16 kHz (ANOVA, F_4,223_ = 20.69, *p* < 0.0001; corrected pair comparisons: *p < 0.05, **p < 0.01, ***p < 0.001). Animals and recording sites: control *n* = 10 and 58; 8 kHz *n* = 5 and 21; 13 kHz *n* = 3 and 18; 8 and 13 kHz *n* = 6 and 41; 16 kHz *n* = 14 and 90. (B) Mean BF at threshold along the tonotopic axis (ANOVA, group F_2,309_ = 2.21; *p* = 0.11; depth F_13,309_ = 8.19, *p* < 0.001). Animals and recording sites: control *n* = 10 and 98; predictable *n* = 14 and 162; and random *n* = 7 and 91. Error bars represent SEM. (C) Left, schematic representation of a sagittal section of the inferior colliculus illustrating the anatomical distribution of the frequency laminas (color lines) and the positioning of the 4 × 4 multielectrode arrays. Right, mean BF along the dorsoventral axis at different rostrocaudal locations for control (dashed lines) and predictable (continuous line) groups (Ro: ANOVA, group F_1,126_ = 5.97, *p* < 0.05; Ca: ANOVA, group F_1,110_ = 4.23, *p* < 0.05). Error bars are omitted for clarity. Animals and recording sites: control *n* = 6 and 277; predictable *n* = 7 and 289. Numerical data for this figure found in S2 Data. BF, best frequency; Ca, caudal; Ce1, central 1; Ce2, central 2; Ro, rostral.
(TIF)

S5 Fig Predictable sound exposure locally modifies SNR and increases behavioral spontaneous frequency generalization. (A) SNR between depth, at which BF matches the exposed frequency and depth with maximum spontaneous activity for animals exposed to 8 kHz or 16 kHz (wilcoxon signed rank test, **p < 0.01, *n* = 12 pairs, 9 exposed to 16 kHz and 3 exposed to 8 kHz). Inset, PSTHs of the responses of an example mouse (gray dots) for the depth with highest spontaneous activity (black) and depth at which BF matched the exposed frequency (pink). (B) Mean intensity threshold for each recording site (ANOVA, F_2,308_ = 0.85, *p* = 0.42). Animals and recording sites: control *n* = 10 and 98; predictable *n* = 14 and 162; random *n* = 7 and 91. (C) Mean latency to the response to the corresponding BF ± 0.25 octaves for the adjacent (left) or tuned (right) regions (left, adjacent ANOVA, group F_2,115_ = 3.51, *p* < 0.05; right, tuned: ANOVA, group F_2,138_ = 4.56, *p* < 0.05). Animals and recording sites: adjacent: control *n* = 10 and 34; predictable *n* = 14 and 60; and random *n* = 7 and 26; tuned: control *n* = 10 and 42; predictable *n* = 14 and 68; and random *n* = 7 and 34. Corrected pair comparisons *p < 0.05, **p < 0.01. (D) Classification accuracy probability for decoded frequencies between 10 and 14 kHz (adjacent region) and 14 and 20 kHz (tuned region) across groups. (ANOVA, group F_2,242_ = 7.33, *p* = 0.0008, range F_1,242_ = 5.75, *p* = 0.017, no interaction F_2,242_ = 2.29, *p* = 0.10). Animals: control *n* = 10; predictable *n* = 14; random *n* = 7. Corrected pair comparisons: *p* = 0.034 predictable versus control, *p* = 0.0005 random versus control. (E) PPI mean and individual discrimination thresholds (ANOVA, group F_2,21_ = 4.32, *p* < 0.05. Corrected pair comparisons: *p < 0.05). Control *n* = 7; predictable *n* = 8; random *n* = 9. Error bars represent SEM. Numerical data for this figure found in S2 Data. BF, best frequency; PPI, prepulse inhibition of the auditory startle reflex; PSTH, peri-stimulus time histogram; SNR, signal-to-noise ratio.
(TIF)

S6 Fig Cortical inactivation subtly increases collicular activity without affecting tuning. (A) Representative color plots showing the simultaneous evoked LFP at different depths in the AC to stimulation with broadband noise at different sound intensities before (top) and 20 minutes after muscimol application over the cortical surface (bottom). The vertical white dashed lines in each subplot represent the duration of the stimulus (100 ms). (B) Mean BF across depths obtained before and after cortical inactivation (ANOVA, group F_1,196_ = 15.06, *p* < 0.01). For B-C: animals and recording sites: control *n* = 7 and 62; predictable *n* = 6 and 64. (C) Mean tuning curves at 70 dB for different depths in the inferior colliculus for control (blue) and predictable (red) group, before (continuous line) and after (dashed lines) cortical inactivation. Error bars represent SEM. Numerical data for this figure found in S2 Data. AC, auditory cortex; BF, best frequency; LFP, local field potential.
(TIF)

S7 Fig Predictable sound exposure does not affect tuning in the cochlear nucleus or the AC. (A-B) Box–whisker plot of evoked spike rates in response to CF and 8 kHz tone bursts of units with (A) CF 6–12kHz and (B) CF 12–24Hz (wilcoxon signed rank test, *p* > 0.5 for all comparisons). (C-D) Analysis of thresholds and sharpness of tuning (Q10dB, CF divided by the bandwidth of threshold tuning curve 10 dB above threshold) of all recorded cochlear nucleus units. Units with CF between 6 and 24 kHz were grouped by CF into 2 octave bins (CF group 6–12kHz and CF group 12–24kHz; unpaired t test, *p* > 0.5 for all comparisons). (E) Mean tuning curves in the primary auditory cortices (A1 and AAF) of recording sites with a BF of 11–20 kHz (ANOVA, group F_2,240_ = 0.17, *p* = 0.086). Control *n* = 5; predictable *n* = 4; random *n* = 4. (F) Mean PSTH of individual-mouse BF evoked at 70 dB. Vertical lines delimit sound duration. Responses were divided in onset (0–30 ms) and late (31–80 ms) (onset, Kruskal-Wallis test, *Χ*^2^_2,88_ = 0.26, *p* = 0.87; late, *Χ*^2^_2,127_ = 3.07, *p* = 0.21). Control *n* = 5; predictable *n* = 4; random *n* = 4. (G) Distribution of cortical BF across all recording sites. Frequency categories are ±0.5 octaves–wide bins. Error bars represent SEM. Numerical data for this figure found in S2 Data. AC, auditory cortex; BF, best frequency; CF, characteristic frequency; PSTH, peri-stimulus time histogram.
(TIF)

S8 Fig Sound exposure results in changes in presynaptic markers and asymmetrical effects on lateral inhibition and facilitation. (A) Representative photomicrographs of an area of the inferior colliculus centered at 300 μm in depth double-labeled for VGAT and Vglut2 for control (upper panels) and predictable (lower panels) groups. Scale bar 10 μm. (B) Quantification of the positive puncta for VGAT and Vglut2 in the dorsal (300 μm) and ventral (600 μm) areas (wilcoxon signed rank test, *p < 0.05, *n* = 7 for each group). Numerical data for this figure found in S2 Data. VGAT, GABA vesicular transporter; Vglut2, glutamate vesicular transporter 2.
(TIF)

S1 Table Quantification of relative gene expression measured by quantitative real-time PCR. (XLSX)

## Abbreviations

AC: auditory cortex
AMPA: α-amino-3-hydroxy-5-methyl-4-isoxazolepropionic acid
AUROCC: area under the ROC curve
BBN: broadband noise
*BDNF*: brain-derived neurotrophic factor
BF: best frequency
CF: characteristic frequency
DiI: 1,1'-dioactedecyl-3,3,3,3'-tethramethyl indocarbocyanide
LI: latent inhibition
PPI: prepulse inhibition of the auditory startle reflex
PSTH: peri-stimulus time histogram
ROC: receiver operating characteristic
Rpl13a: ribosomal protein L13a
SNR: signal-to-noise ratio
VGAT: GABA vesicular transporter
VGLUT2: glutamate vesicular transporter 2
