# Supplementary figures and images for: Auditory midbrain coding of statistical learning that results from discontinuous sensory stimulation
Source: PLoS Biol. 2018 Jul 26;16(7):e2005114. doi: 10.1371/journal.pbio.2005114 (PMC6065201; doi:10.1371/journal.pbio.2005114)

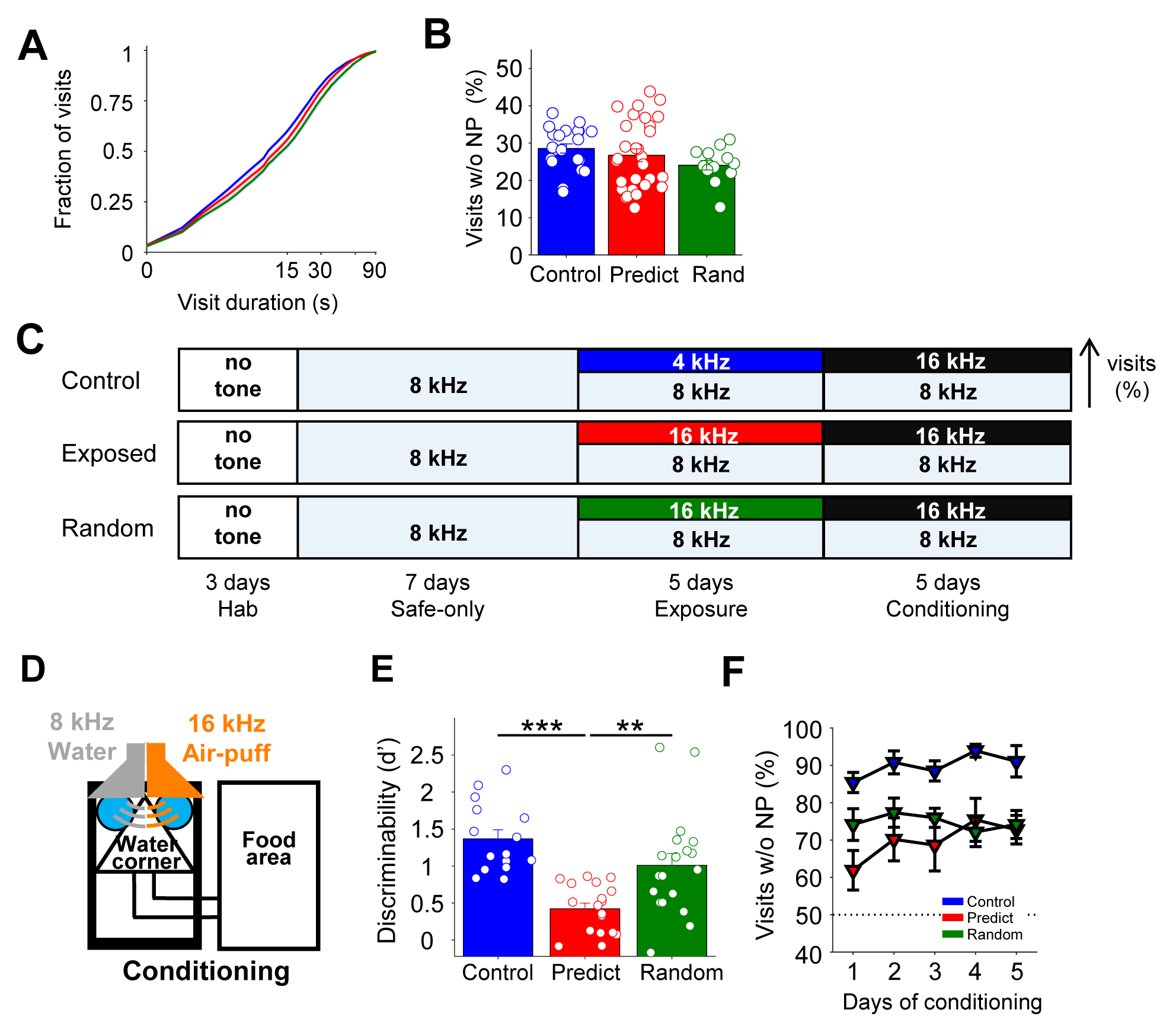

Supplement: S1 Fig — (A) Cumulative distribution of the visit duration to the water corner area. (B) Mean daily percentage of visits without NPs was similar between groups (ANOVA, F2,60 = 1.47, p = 0.23). (C) Scheme of the latent inhibition protocol. All phases were identical across groups except for the exposure phase. Colored boxes indicate the frequency of sound exposure (30% of visits). To avoid sound novelty effects, 8 kHz was used in remaining visits (safe visits). Conditioning took place only during the conditioning phase and only in visits in which 16 kHz was played (black bars). (D) Schematic representation of the conditioning phase in the latent inhibition paradigm. Safe visits were accompanied by an 8 kHz tone (left, gray color). Conditioned visits were accompanied by a 16 kHz sound, and NP on either side resulted in an air puff (right, orange color). (E) Mean discriminability index (d’) during the first day of conditioning was lower for the predictable group (ANOVA, F2,49 = 13.69, p < 0.01; ***p < 0.0001; **p < 0.001). Control n = 15; predictable n = 18; random n = 19. (F) Mean visits without NPs per day and group during the first 5 days of conditioning for the conditioning visits only. Error bars represent SEM. Numerical data for this figure found in S2 Data. NP, nose-poke. (TIF) [file pbio.2005114.s003.tif]

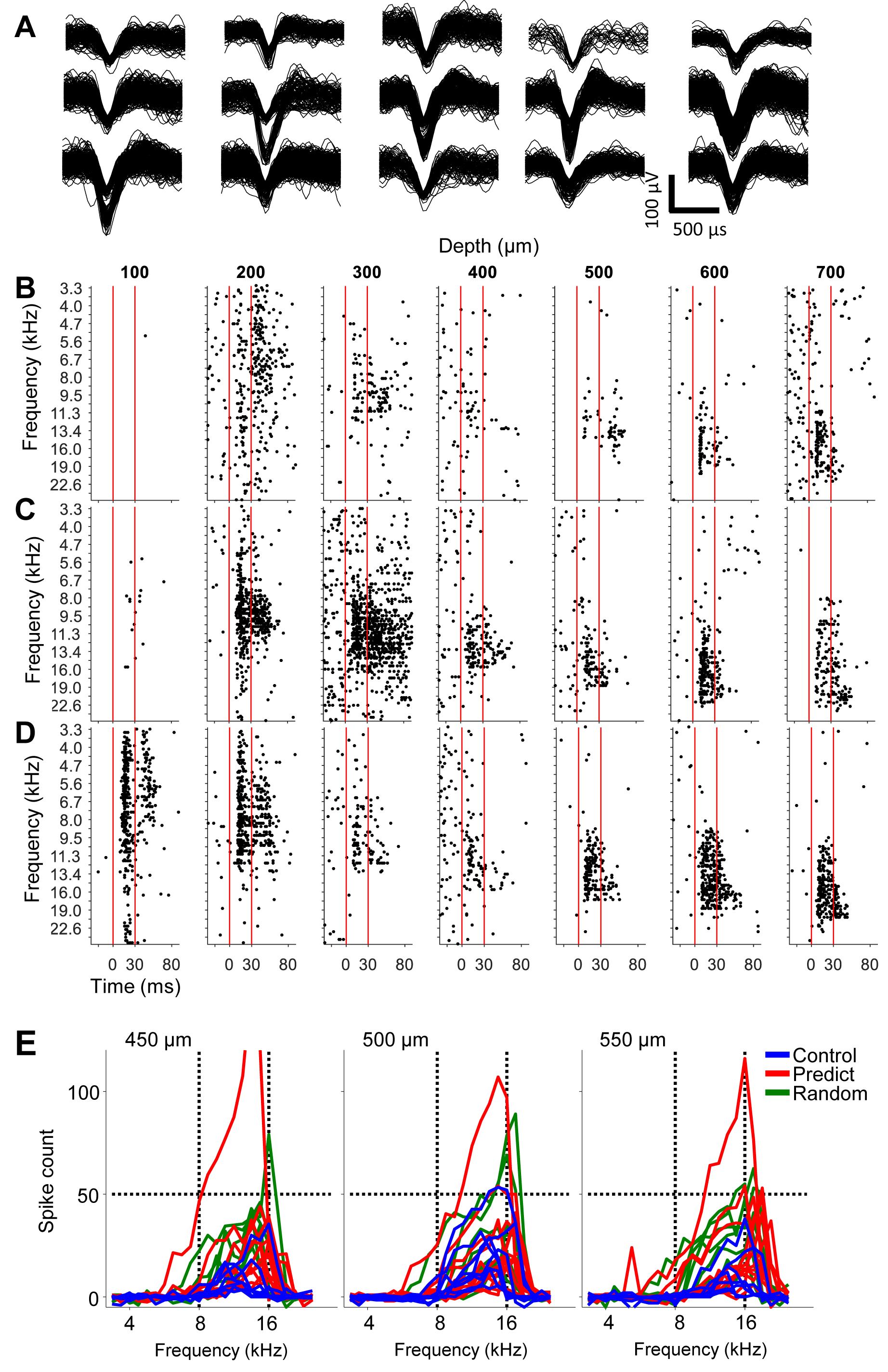

Supplement: S2 Fig — (A) Representative examples of spike waveforms recorded from a given electrode at a given depth (300–600 μm) for the control (upper row), predictable (middle row), and random (lower row) groups. (B-D) Representative examples of raster plots recorded at 70 dB SPL at different depths from one control (B), one predictable (C), and one random animal (D). Each dot represents a spike and each line, one of 5 repetitions of a 30 ms tone. Vertical red lines indicate the onset and offset of the tone. (E) Individual tuning curves for animals in the control (blue), predictable (red), and random (green) groups for depths with BF of 16 kHz in the mice in the predictable and random groups. Numerical data for this figure found in S2 Data. (TIF) [file pbio.2005114.s004.tif]

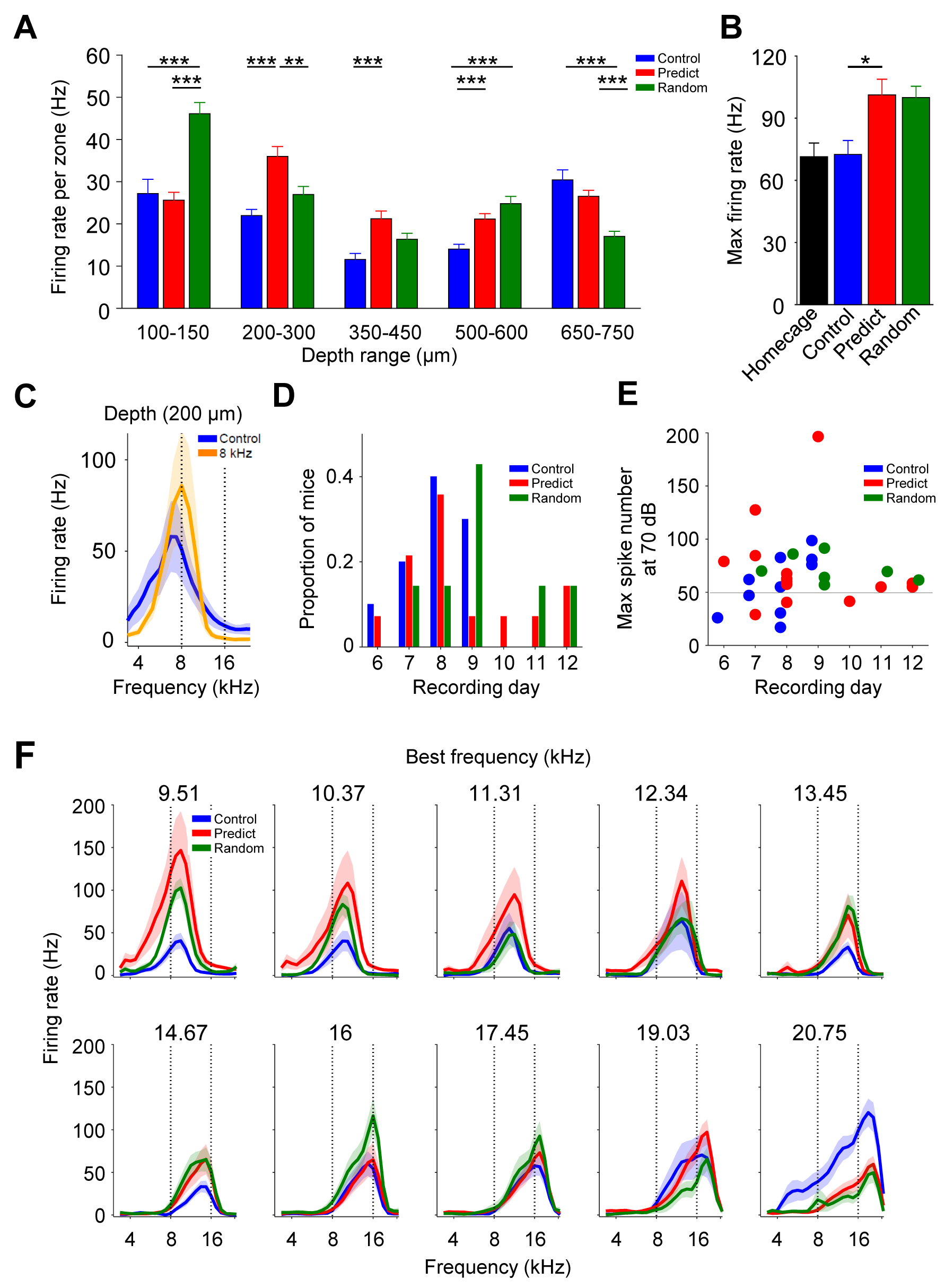

Supplement: S3 Fig — (A) Mean firing rate per collicular zone (100–150 μm: ANOVA, group F2,1080 = 22.64, p < 0.0001; 200–300 μm: ANOVA, group F2,1944 = 15.21, p < 0.001; 350–450 μm: ANOVA, group F2,1680 = 9.54, p < 0.001; 500–600 μm: ANOVA, group F2,1848 = 21.46, p < 0.0001; 650–750 μm: ANOVA, group F2,1512 = 21.31, p < 0.0001. Corrected pair comparisons ***p < 0.0001, **p < 0.01). For A-B, D-E: animals and recording sites: home cage n = 6 and 72; control n = 10 and 98; predictable n = 14 and 162; random n = 7 and 91. (B) Mean maximum firing rate (ANOVA, group F3,367 = 4.2, p < 0.01; corrected pair comparisons: *p < 0.05). (C) Group mean tuning curves of responses at 200 μm of animals in control and 8 kHz–exposed predictable group (ANOVA, group × frequency interaction F23,276 = 4.22, p < 0.05). Control n = 10; 8 kHz n = 4. (D) Distribution of number of days in the Audiobox per group. Animals: control n = 10; predictable n = 14; random n = 7. (E) Peak response per mouse across days in the Audiobox per group as in (D). Overall ANOVA (group × days) revealed no effect of group F2,22 = 1.63, p = 0.22; or days F6,22 = 1.44, p = 0.25. There was no effect of days within each group: control, F3,6 = 2.62, p = 0.15; predictable, F6,7 = 3.06, p = 0.08; random, F4,2 < 1. (F) Tuning curves aligned by BF ± 0.05% for BFs with at least 4 mice/group. An overall ANOVA (group × tuning BF × frequency played) revealed an effect of group F2,4752 = 12.55, p < 0.001; BF F9,4752 = 10.01, p < 0.001; and frequency F23,4752 = 40.96, p < 0.001; and an interaction between group and BF F18,4752 = 12.81, p < 0.001; and BF and frequency F207,4752 = 5.21, p < 0.001. Within each BF range, all group comparisons revealed an effect of group: 9,510 Hz, ANOVA, group F2,384 = 15.46, p < 0.001; 10,370 Hz, ANOVA, group F2,456 = 4.91, p < 0.001; 11,310 Hz, ANOVA, group F2,504 = 4.98, p < 0.01; 12,340 Hz, ANOVA, group F2,336 = 1.51, p = 0.22; 13,450 Hz, ANOVA, group F2,528 = 7.54, p < 0.001; 14,670 Hz, ANOVA, group F2,456 = 8.15, p [file pbio.2005114.s005.tif]

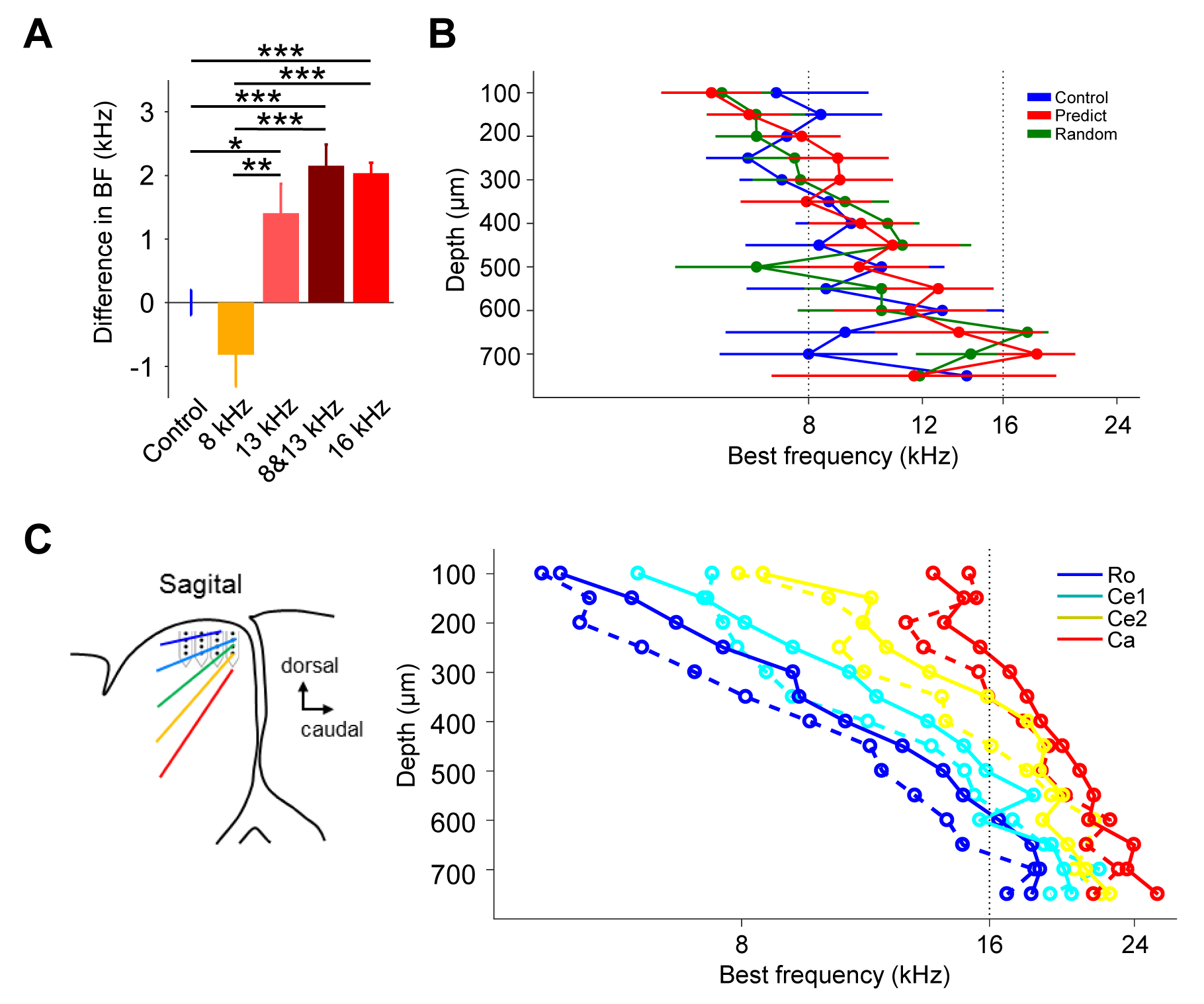

Supplement: S4 Fig — (A) Across depth mean difference in BF with respect to mean BF of control group for groups exposed to frequencies other than 16 kHz (ANOVA, F4,223 = 20.69, p < 0.0001; corrected pair comparisons: *p < 0.05, **p < 0.01, ***p < 0.001). Animals and recording sites: control n = 10 and 58; 8 kHz n = 5 and 21; 13 kHz n = 3 and 18; 8 and 13 kHz n = 6 and 41; 16 kHz n = 14 and 90. (B) Mean BF at threshold along the tonotopic axis (ANOVA, group F2,309 = 2.21; p = 0.11; depth F13,309 = 8.19, p < 0.001). Animals and recording sites: control n = 10 and 98; predictable n = 14 and 162; and random n = 7 and 91. Error bars represent SEM. (C) Left, schematic representation of a sagittal section of the inferior colliculus illustrating the anatomical distribution of the frequency laminas (color lines) and the positioning of the 4 × 4 multielectrode arrays. Right, mean BF along the dorsoventral axis at different rostrocaudal locations for control (dashed lines) and predictable (continuous line) groups (Ro: ANOVA, group F1,126 = 5.97, p < 0.05; Ca: ANOVA, group F1,110 = 4.23, p < 0.05). Error bars are omitted for clarity. Animals and recording sites: control n = 6 and 277; predictable n = 7 and 289. Numerical data for this figure found in S2 Data. BF, best frequency; Ca, caudal; Ce1, central 1; Ce2, central 2; Ro, rostral. (TIF) [file pbio.2005114.s006.tif]

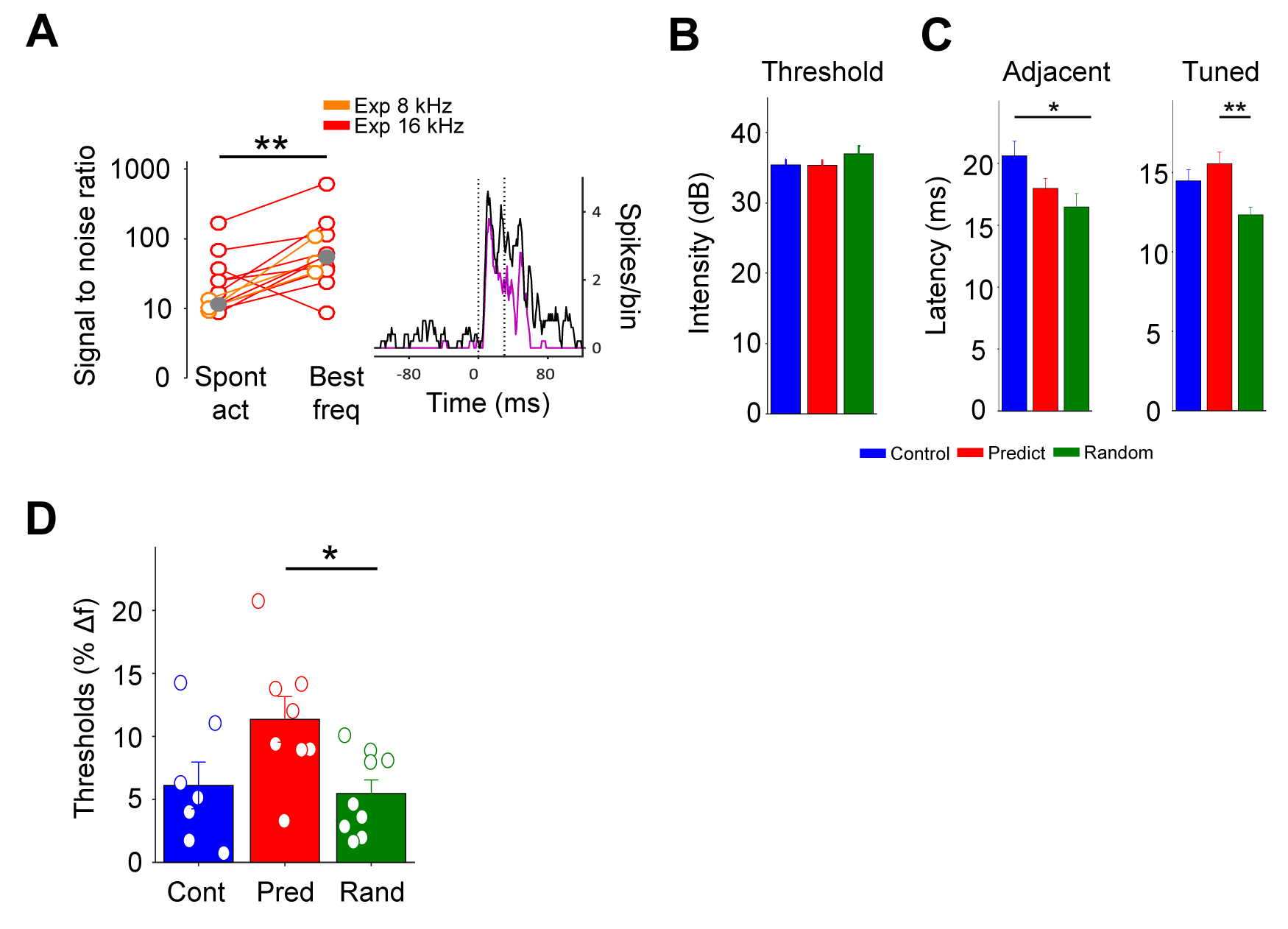

Supplement: S5 Fig — (A) SNR between depth, at which BF matches the exposed frequency and depth with maximum spontaneous activity for animals exposed to 8 kHz or 16 kHz (wilcoxon signed rank test, **p < 0.01, n = 12 pairs, 9 exposed to 16 kHz and 3 exposed to 8 kHz). Inset, PSTHs of the responses of an example mouse (gray dots) for the depth with highest spontaneous activity (black) and depth at which BF matched the exposed frequency (pink). (B) Mean intensity threshold for each recording site (ANOVA, F2,308 = 0.85, p = 0.42). Animals and recording sites: control n = 10 and 98; predictable n = 14 and 162; random n = 7 and 91. (C) Mean latency to the response to the corresponding BF ± 0.25 octaves for the adjacent (left) or tuned (right) regions (left, adjacent ANOVA, group F2,115 = 3.51, p < 0.05; right, tuned: ANOVA, group F2,138 = 4.56, p < 0.05). Animals and recording sites: adjacent: control n = 10 and 34; predictable n = 14 and 60; and random n = 7 and 26; tuned: control n = 10 and 42; predictable n = 14 and 68; and random n = 7 and 34. Corrected pair comparisons *p < 0.05, **p < 0.01. (D) Classification accuracy probability for decoded frequencies between 10 and 14 kHz (adjacent region) and 14 and 20 kHz (tuned region) across groups. (ANOVA, group F2,242 = 7.33, p = 0.0008, range F1,242 = 5.75, p = 0.017, no interaction F2,242 = 2.29, p = 0.10). Animals: control n = 10; predictable n = 14; random n = 7. Corrected pair comparisons: p = 0.034 predictable versus control, p = 0.0005 random versus control. (E) PPI mean and individual discrimination thresholds (ANOVA, group F2,21 = 4.32, p < 0.05. Corrected pair comparisons: *p < 0.05). Control n = 7; predictable n = 8; random n = 9. Error bars represent SEM. Numerical data for this figure found in S2 Data. BF, best frequency; PPI, prepulse inhibition of the auditory startle reflex; PSTH, peri-stimulus time histogram; SNR, signal-to-noise ratio. (TIF) [file pbio.2005114.s007.tif]

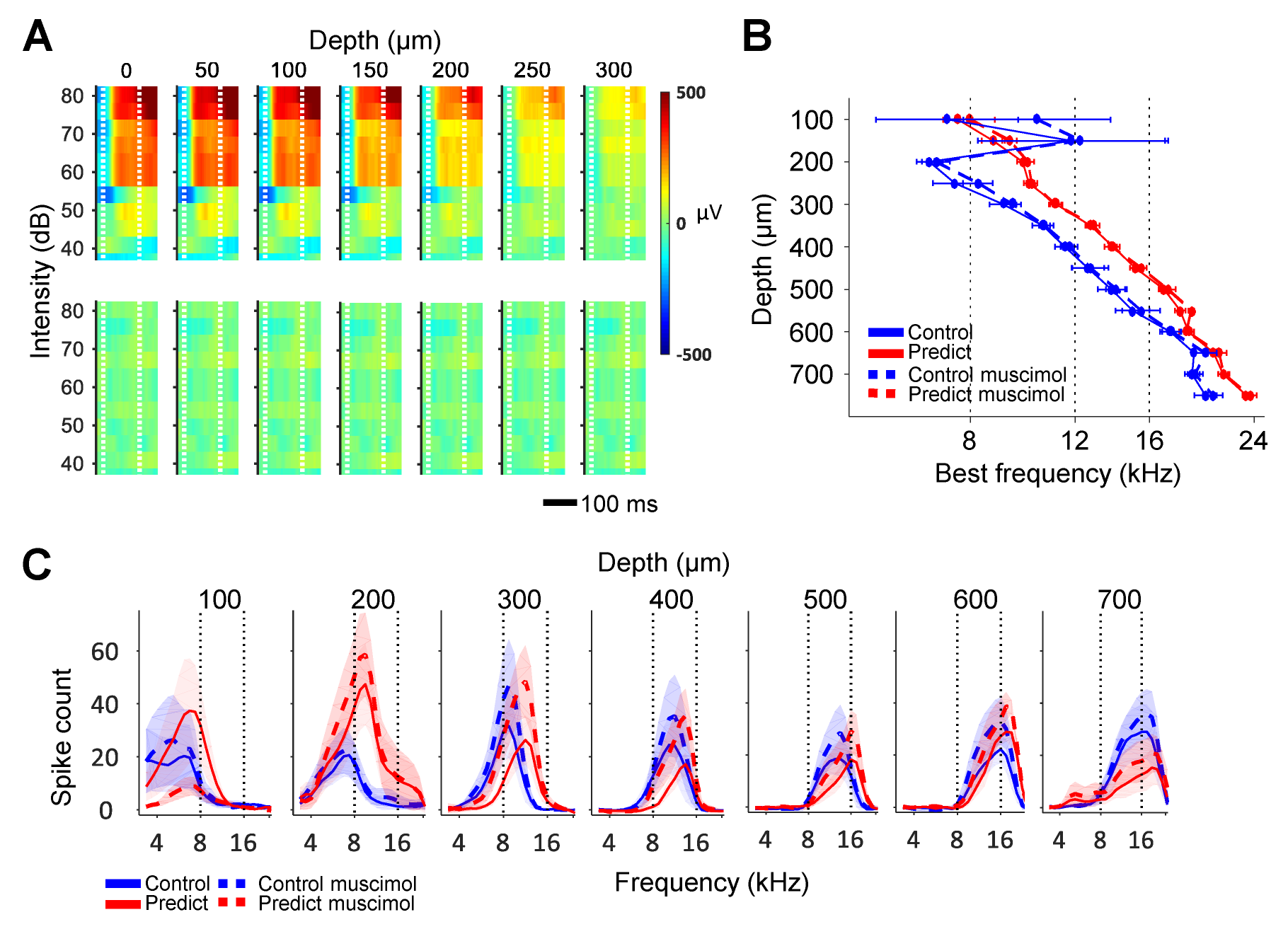

Supplement: S6 Fig — (A) Representative color plots showing the simultaneous evoked LFP at different depths in the AC to stimulation with broadband noise at different sound intensities before (top) and 20 minutes after muscimol application over the cortical surface (bottom). The vertical white dashed lines in each subplot represent the duration of the stimulus (100 ms). (B) Mean BF across depths obtained before and after cortical inactivation (ANOVA, group F1,196 = 15.06, p < 0.01). For B-C: animals and recording sites: control n = 7 and 62; predictable n = 6 and 64. (C) Mean tuning curves at 70 dB for different depths in the inferior colliculus for control (blue) and predictable (red) group, before (continuous line) and after (dashed lines) cortical inactivation. Error bars represent SEM. Numerical data for this figure found in S2 Data. AC, auditory cortex; BF, best frequency; LFP, local field potential. (TIF) [file pbio.2005114.s008.tif]

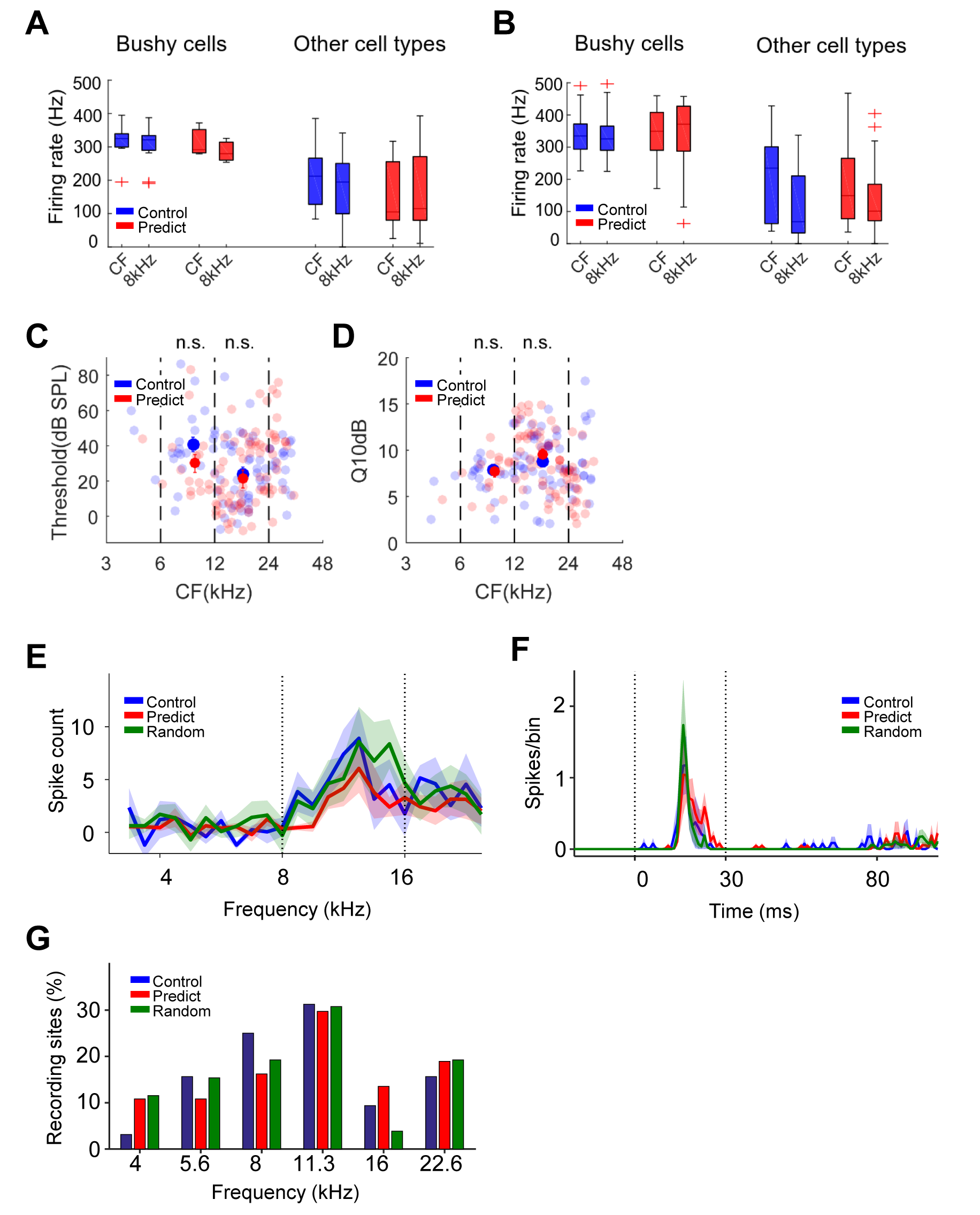

Supplement: S7 Fig — (A-B) Box–whisker plot of evoked spike rates in response to CF and 8 kHz tone bursts of units with (A) CF 6–12kHz and (B) CF 12–24Hz (wilcoxon signed rank test, p > 0.5 for all comparisons). (C-D) Analysis of thresholds and sharpness of tuning (Q10dB, CF divided by the bandwidth of threshold tuning curve 10 dB above threshold) of all recorded cochlear nucleus units. Units with CF between 6 and 24 kHz were grouped by CF into 2 octave bins (CF group 6–12kHz and CF group 12–24kHz; unpaired t test, p > 0.5 for all comparisons). (E) Mean tuning curves in the primary auditory cortices (A1 and AAF) of recording sites with a BF of 11–20 kHz (ANOVA, group F2,240 = 0.17, p = 0.086). Control n = 5; predictable n = 4; random n = 4. (F) Mean PSTH of individual-mouse BF evoked at 70 dB. Vertical lines delimit sound duration. Responses were divided in onset (0–30 ms) and late (31–80 ms) (onset, Kruskal-Wallis test, Χ22,88 = 0.26, p = 0.87; late, Χ22,127 = 3.07, p = 0.21). Control n = 5; predictable n = 4; random n = 4. (G) Distribution of cortical BF across all recording sites. Frequency categories are ±0.5 octaves–wide bins. Error bars represent SEM. Numerical data for this figure found in S2 Data. AC, auditory cortex; BF, best frequency; CF, characteristic frequency; PSTH, peri-stimulus time histogram. (TIF) [file pbio.2005114.s009.tif]

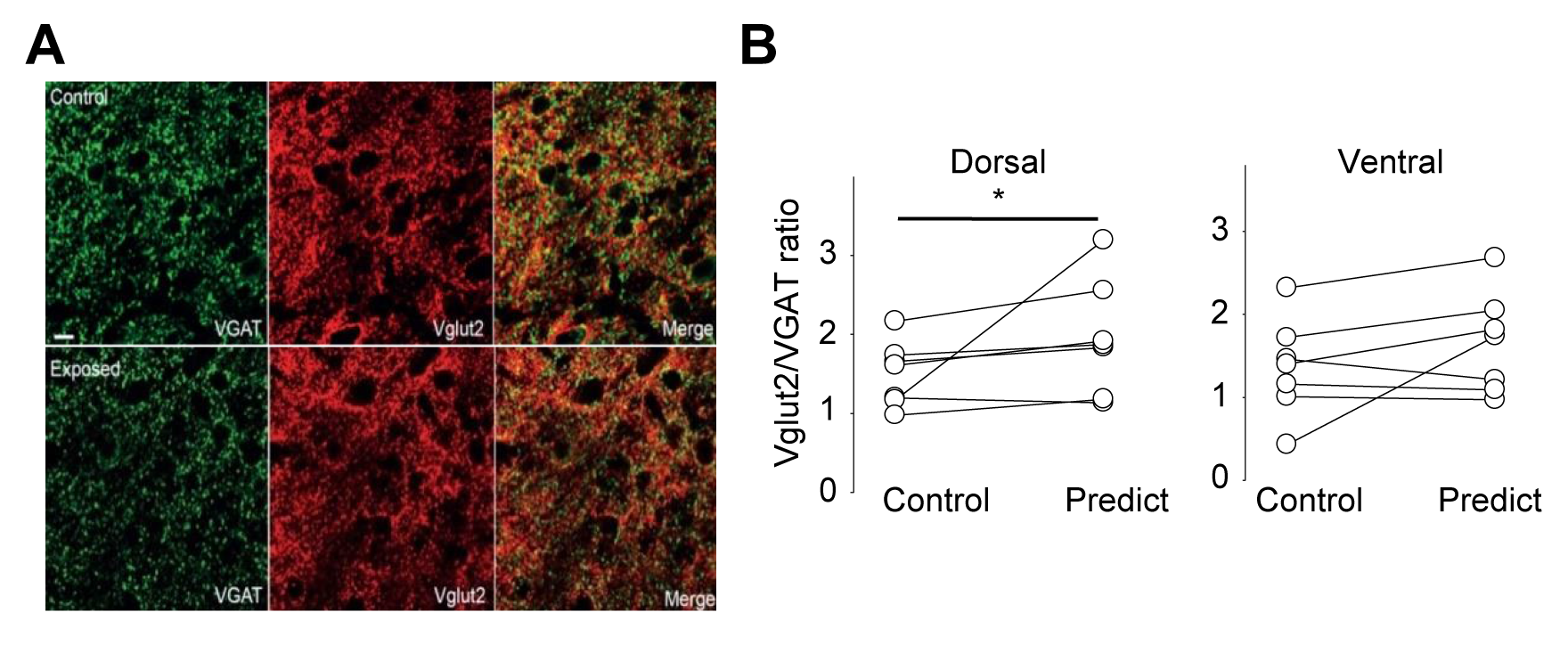

Supplement: S8 Fig — (A) Representative photomicrographs of an area of the inferior colliculus centered at 300 μm in depth double-labeled for VGAT and Vglut2 for control (upper panels) and predictable (lower panels) groups. Scale bar 10 μm. (B) Quantification of the positive puncta for VGAT and Vglut2 in the dorsal (300 μm) and ventral (600 μm) areas (wilcoxon signed rank test, *p < 0.05, n = 7 for each group). Numerical data for this figure found in S2 Data. VGAT, GABA vesicular transporter; Vglut2, glutamate vesicular transporter 2. (TIF) [file pbio.2005114.s010.tif]
